# GCDH Acetylation Orchestrates DNA Damage Response and Autophagy via Mitochondrial ROS to Suppress Hepatocellular Carcinoma Progression

**DOI:** 10.34133/research.0862

**Published:** 2025-08-29

**Authors:** Wei Tian, Yue Yang, Lili Meng, Chao Ge, Yuqi Liu, Canxue Zhang, Zhihong Huang, Chi Zhang, Hua Tian

**Affiliations:** ^1^ State Key Laboratory of Systems Medicine for Cancer, Shanghai Cancer Institute, Renji Hospital, Shanghai Jiao Tong University School of Medicine, Shanghai, China.; ^2^ Department of Pathology, Zhongshan Hospital, Fudan University, Shanghai, China.; ^3^ Department of Pathology, The Affiliated Hospital of Youjiang Medical University for Nationalities, Baise, China.; ^4^ The Key Laboratory of Molecular Pathology (Hepatobiliary Diseases) of Guangxi, Baise, China.

## Abstract

Metabolic enzyme dysregulation promotes hepatocellular carcinoma (HCC) progression through metabolic reprogramming and lysine acetylation. Glutaryl-CoA dehydrogenase (GCDH), a key enzyme in lysine metabolism, has been demonstrated to play an essential role in modulating lysine crotonylation, which impacts the progression of HCC. However, the specific mechanisms by which GCDH influences lysine acetylation in HCC have not been completely clarified. In this study, GCDH was found to be acetylated at lysine 438 by acetyltransferase P300 and deacetylated by HDAC1. GCDH K438 acetylation was critical for its tumor-suppressive function in HCC cells. Overexpression of GCDH led to elevated levels of reactive oxygen species (ROS) and reduced oxidative phosphorylation (OXPHOS), thereby triggering ATR/Chk1-mediated DNA damage repair dysfunction and promoting autophagy in HCC cells. Furthermore, our investigation demonstrated that decreased GCDH expression was markedly associated with shorter overall survival in HCC patients and served as an independent prognostic indicator. Collectively, our findings demonstrate that the acetylation of GCDH at lysine 438 (K438), mediated by P300 and HDAC1, plays a vital role in the tumor-suppressive activities of HCC cells. GCDH inhibits HCC progression through ROS-mediated DNA repair dysfunction and autophagy.

## Introduction

Hepatocellular carcinoma (HCC) constitutes about 90% of all primary liver malignancies. Approximately 25% to 70% of individuals with HCC are identified in the later stages of the disease, which is associated with an unfavorable prognosis. Multikinase inhibitors and immune checkpoint inhibitors are now recognized as the standard therapies for advanced HCC [1]. However, the treatment has only achieved limited clinical benefit. Therefore, there is an urgent need to gain a comprehension of the molecular mechanisms driving HCC progression for the purpose of identifying prospective therapeutic targets.

Metabolic reprogramming is a hallmark of cancer cells, endowing them with enhanced metabolic plasticity [2]. Lysine and tryptophan, both of which are essential amino acids, are indispensable for the survival of cancer cells [3]. The metabolism of lysine and tryptophan occurs through a shared pathway. The dehydrogenase E1 and transketolase domain containing 1 (DHTKD1) facilitates the conversion of lysine and tryptophan into the metabolite glutaryl coenzyme A (CoA). Following the action of glutaryl-CoA dehydrogenase (GCDH), glutaryl CoA is converted into crotonyl CoA, which is subsequently processed to acetyl CoA and enters the tricarboxylic acid (TCA) cycle [4]. Defects in the gene encoding GCDH lead to amino acid metabolism disorders, resulting in the accumulation of metabolic products in the body and leading to type 1 glutaric aciduria, a rare inherited metabolic disorder [5]. Emerging evidence indicates that GCDH facilitates the conversion of glutaryl-CoA into crotonyl-CoA, thereby promoting lysine crotonylation (Kcr), which exhibits potential antitumor activity [6]. Additionally, recent studies have revealed that glioblastoma stem cells (GSCs) undergo lysine catabolism reprogramming by up-regulating GCDH, resulting in the buildup of intracellular crotonyl-CoA and the modification of histone H4 lysine through crotonylation [4]. Moreover, knockdown of GCDH in melanoma cells led to the activation of cell death programs, which could be prevented by inhibiting the upstream lysine catabolism enzyme DHTKD1 [7]. These findings highlight the essential role of GCDH in regulating lysine metabolism through its influence on lysine crotonylation. By modulating this posttranslational modification (PTM), GCDH exerts significant control over metabolic pathways that are essential for the initiation and progression of malignant tumors.

Lysine acetylation serves as a general PTM that affects histone as well as nonhistone proteins, contributing to the oversight of a broad spectrum of cellular processes, such as metabolic reprogramming [8]. There is a significant connection between histone acetylation and tumorigenesis, which influences multiple biological functions of tumor cells, such as proliferation [9], metastasis [10], and stemness [11]. In addition to its role in modulating enzyme efficiency and stability, lysine acetylation has been recognized as a factor in the regulation of gene expression [12].

Although current studies have highlighted the critical role of histone acetylation in regulating tumorigenesis, tumor progression, and tumor-associated microenvironment, the regulatory mechanisms and functional implications of the GCDH acetylation remain largely unexplored, especially in HCC. In this study, we evaluated the acetylation mechanism by which GCDH is regulated, and explored the impact and underlying biological mechanisms of GCDH acetylation in HCC progression.

## Results

### GCDH is acetylated by P300

**Fig. 1.**
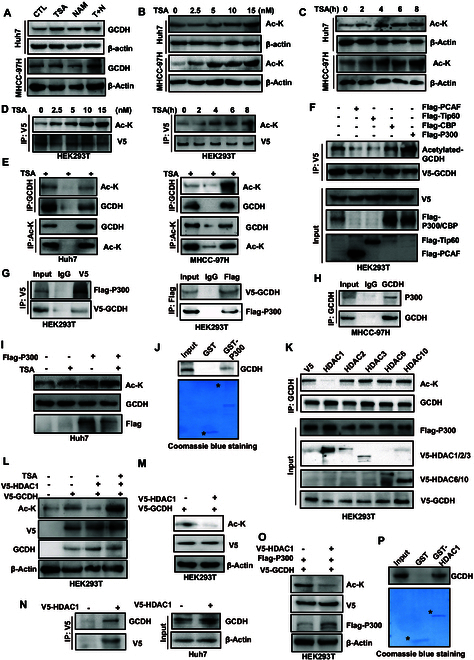
GCDH is acetylated by P300 and deacetylated by HDAC1. (A) Huh7 and MHCC-97H cells were treated with 3 μM TSA and 5 mM NAM for 12 h. GCDH expression was detected by WB. (B and C) Acetylation levels of GCDH were increased upon TSA treatment in a dose-dependent (B) and time-dependent (C) manner. (D) HEK293T cells expressing V5-GCDH were treated with TSA, followed by immunoprecipitation with anti-V5 antibody and detection with pan-acetyl-lysine antibody. (E) TSA treatment enhanced GCDH acetylation in Huh7 and MHCC-97H cells. (F) V5-GCDH was coexpressed with diverse acetyltransferases in HEK293T cells. Expression of GCDH acetylation was detected. (G and H) Co-immunoprecipitation (IP) assays confirmed the interaction between Flag-P300 and V5-GCDH in both HEK293T (G) and MHCC-97H (H) cells. (I) GCDH acetylation is increased in the presence of TSA (3 μM) for 12 h in Huh7 cells transfected with Flag-P300. (J) The interaction between GCDH and P300 was confirmed by GST pull-down assay. (K) V5-GCDH was cotransfected with Flag-P300 and diverse HDAC plasmids in HEK293T cells, and cell lysates were detected by SDS-PAGE and immunoblotting with indicated antibodies. (L) HEK293T cells cotransfected with V5-GCDH and V5-HDAC1 were treated with or without 3 μM TSA for 12 h before harvesting, followed by immunoblotting. (M and N) V5-GCDH, together with V5-HDAC1, was transfected in HEK293T (M) and Huh7 (N) cells. Lysates were immunoblotted with indicated antibodies. (O) V5-GCDH was cotransfected with Flag-P300 and V5-HDAC1 plasmids in HEK293T cells, and cell lysates were detected by SDS-PAGE and immunoblotting with indicated antibodies. (P) The interaction between GCDH and HDAC1 was confirmed by GST pull-down assay.

To investigate whether GCDH is modified by acetylation, Huh7 and MHCC-97H cells were exposed to trichostatin A (TSA), a classical histone deacetylase (HDAC) inhibitor, in conjunction with nicotinamide (NAM), which inhibits SIRT family members, to obstruct the activity of lysine deacetylases (KDACs) throughout the procedure. The results revealed that TSA treatment elevated endogenous GCDH acetylation, whereas NAM had no such effect (Fig. 1A), suggesting a potential role for classical HDAC family members in the deacetylation of GCDH. Consistently, treatment with TSA markedly enhanced acetylation of GCDH in a dose- and time-dependent manner (Fig. 1B to D). We conducted immunoprecipitation with an acetylated-lysine (Ac-K) antibody and subsequently detected GCDH using a specific antibody. The analysis confirmed that endogenous GCDH possesses the ability to undergo acetylation (Fig. 1E).

To identify the specific acetyltransferase responsible for GCDH acetylation, we cotransfected GCDH with various acetyltransferases, including PCAF, Tip60, CBP, and P300. The results indicated that P300 was the only acetyltransferase capable of markedly enhancing GCDH acetylation (Fig. 1F). Given that GCDH was acetylated by P300, we sought to determine whether GCDH interacts with P300. We found that exogenous GCDH were coimmunoprecipitated with Flag-P300 in HEK293T cells (Fig. 1G). The endogenous interaction between GCDH and P300 was confirmed in MHCC-97H cells (Fig. 1H). Moreover, Huh7 cells were transfected with Flag-P300 and subsequently treated with TSA for 12 h. The results revealed that TSA enhanced GCDH acetylation (Fig. 1I). Furthermore, glutathione *S*-transferase (GST) pull-down assays demonstrated that GST-P300 directly interacts with GCDH, indicating a physical binding interaction between the 2 proteins (Fig. 1J). Taken together, GCDH can be acetylated by P300 both in vivo and in vitro.

### GCDH is deacetylated by HDAC1

The process of acetylation is highly dynamic, and specific KDAC(s) can reverse deacetylation. We therefore investigated potential deacetylases of GCDH. The acetylation level of GCDH was substantially raised after treatment with TSA, whereas it remained unchanged with NAM, suggesting that HDACs may play a role in the deacetylation of GCDH. We then cotransfected V5-GCDH and Flag-P300 together with individual V5-tagged HDACs (HDAC1, HDAC2, HDAC3, HDAC6, and HDAC10) into HEK293T cells. Our results indicated that HDAC1 markedly decreased the acetylation of GCDH (Fig. 1K). Furthermore, the deacetylation of GCDH by HDAC1 could be prevented by TSA treatment in HEK293T cells (Fig. 1L). To examine the interaction between GCDH and HDAC1, we coexpressed V5-GCDH and V5-HDAC1 in HEK293T cells and observed that exogenous HDAC1 could be coimmunoprecipitated with GCDH (Fig. 1M). Additionally, following transfection of Huh7 cells with V5-HDAC1 and subsequent co-immunoprecipitation analysis, the results indicated an interaction between endogenous GCDH and V5-HDAC1 (Fig. 1N). Moreover, the deacetylation of GCDH by HDAC1 was confirmed in HEK293T cells transfected with V5-HDAC1, Flag-P300, and V5-GCDH (Fig. 1O). In addition, the interaction between GCDH and HDAC1 was confirmed by GST pull-down assay (Fig. 1P). Therefore, these findings indicate that HDAC1 is the principal deacetylase responsible for GCDH deacetylation and is capable of directly interacting with GCDH both in vivo and in vitro.

### GCDH is acetylated at residue K438

**Fig. 2.**
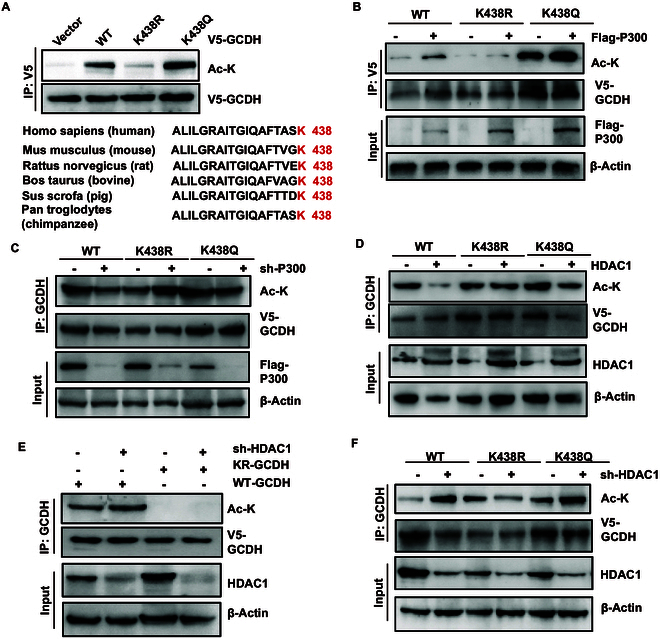
GCDH is acetylated at the evolutionarily conserved lysine 438 (K438). (A) Acetylation of GCDH WT, K438R, or K438Q was detected by pan-acetyl-lysine antibody. GCDH K438 is evolutionarily conserved. Sequence alignment shows that K438 is conserved across 6 species (highlighted in red). (B) P300 enhances K438 acetylation of GCDH. (C) Knockdown of P300 decreases K438 acetylation levels of GCDH. Lysates from P300 WT or knockdown cells were immunoblotted. (D) HDAC1 decreases acetylation of GCDH in HEK293T cells. (E) Knockdown of HDAC1 increases acetylation levels of GCDH WT, not K438R mutation. (F) Knockdown of HDAC1 increases K438 acetylation levels of GCDH in HEK293T cells.

In order to determine the acetylation site of GCDH, we initially explored publicly available PTM databases, such as database for PTM (dbPTM) [13], protein lysine modification database (PLMD) [14], PhosphoSitePlus [15], and acetylation set enrichment based (ASEB) program [16], and located only one potential acetylation site (K438) (Fig. S1A). A protein sequence alignment of GCDH homologs from various species revealed that K438 is evolutionarily conserved in mammals (Fig. 2A). To determine whether the K438 residue is the primary acetylation site, we generated a non-acetylatable mutant by substituting lysine (K) with arginine (R), mimicking acetylation-deficient conditions [17], and assessed the acetylation status of both wild-type (WT) and K438R GCDH. Mutation of Lys^438^ to arginine markedly reduced GCDH acetylation, confirming that this residue is a major acetylation site (Fig. 2A). Subsequently, V5-tagged GCDH-WT, K438R (non-acetylatable), and K438Q (acetylation-mimicking) mutants were cotransfected with Flag-P300 to evaluate the impact of K438 acetylation on GCDH modification and function. We found that the K438R mutation reduced GCDH acetylation, whereas the K438Q mutation markedly enhanced it, confirming that Lys^438^ is a critical acetylation site on GCDH (Fig. 2B). GCDH K438Q acetylation was rapidly enhanced following P300 overexpression, whereas P300 overexpression had no marked effect on acetylation of the K438R mutant. Notably, P300 knockdown reduced K438 acetylation in the K438Q mutant but not in the K438R mutant, further confirming that P300 specifically targets Lys^438^ for acetylation (Fig. 2B and C and Fig. S1B).

To identify the deacetylase responsible for targeting Lys^438^ on GCDH, we assessed the role of HDAC1 in GCDH deacetylation. Notably, HDAC1 overexpression resulted in a more pronounced reduction in acetylation of the K438Q mutant compared to the K438R mutant (Fig. 2D), suggesting that HDAC1 preferentially mediates deacetylation of GCDH at the Lys^438^ residue. To further reinforce the importance of HDAC1 in regulating GCDH acetylation, we employed short hairpin RNA (shRNA) to specifically knock down HDAC1 expression. Our results showed that shHDAC1 treatment led to a marked increase in GCDH acetylation, particularly at the K438 residue. This enhancement was evident in both WT GCDH and the K438Q mutant (Fig. 2E and F and Fig. S1C). Notably, this effect was absent in cells expressing the K438R mutant (Fig. 2F), indicating that lysine 438 is the primary acetylation site. Collectively, these findings demonstrate that K438 is the primary acetylation site of GCDH in HCC and is conserved across various species.

### GCDH inhibits proliferation and metastasis of HCC cells

**Fig. 3.**
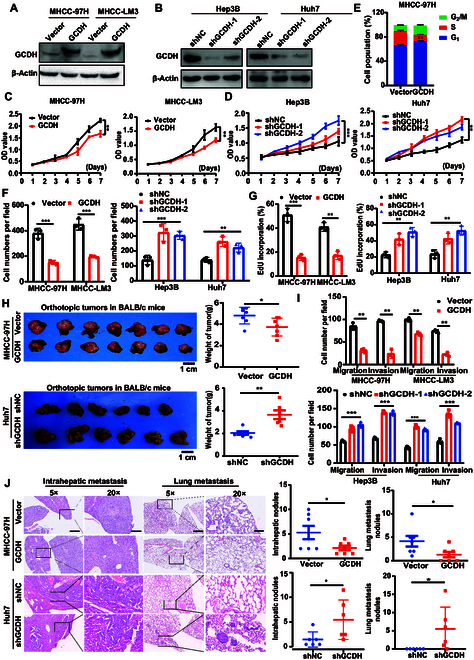
GCDH promotes proliferation, migration, and invasion of HCC cells in vitro and in vivo. (A and B) GCDH expression levels were determined by WB in HCC cells with stable overexpression (A) or knockdown (B). (C and D) The effect of GCDH overexpression (C) or knockdown (D) on HCC cell proliferation was assessed by a CCK-8 assay. (E) The cell cycle distribution of cells was analyzed by flow cytometry. (F and G) The effect of GCDH on HCC cell proliferation was assessed by colony formation assay (F) and EdU assay (G). (H) Xenograft tumor models using MHCC-97H and Huh7 cells with GCDH overexpression or knockdown. Liver weights were quantified (right panel). (I) The effects of GCDH overexpression and knockdown on HCC cell migration and invasion were assessed by transwell assays. (J) Representative images of intrahepatic nodules and lung nodules formed by GCDH-modulated HCC cells. Quantification is shown on the right. Scale bar, 50 μm. * *P* < 0.05; ** *P* < 0.01; *** *P* < 0.001.

MHCC-LM3, MHCC-97H, Hep3B, and Huh7 cells were selected for gain- and loss-of-function studies based on their endogenous GCDH expression (Fig. 3A and B and Fig. S2). GCDH overexpression inhibited HCC cell proliferation, induced G_1_ arrest, suppressed colony formation, and reduced 5-ethynyl-2′-deoxyuridine (EdU) incorporation, while GCDH knockdown had opposing effects (Fig. 3C to G and Fig. S3A to F), highlighting its antiproliferative role in HCC.

In vivo tumor xenograft assays showed that GCDH overexpression markedly inhibited HCC tumor growth, whereas GCDH knockdown markedly enhanced tumor development (Fig. 3H). Consistent with these findings, elevated levels of proliferating cell nuclear antigen (PCNA) were detected in tumor tissues from the GCDH knockdown group compared to the GCDH-overexpressing group (Fig. S3G and H), further supporting a role for GCDH in suppressing HCC cell proliferation.

Transwell assays demonstrated that GCDH overexpression markedly suppressed cell migration and invasion, whereas GCDH knockdown markedly enhanced these processes (Fig. 3I and Fig. S3I to K). Histopathological examination of xenografted mice further showed that tumors derived from GCDH-silenced cells formed more intrahepatic and lung metastases than control tumors, whereas GCDH overexpression led to a marked reduction in metastatic spread (Fig. 3J). Collectively, these findings indicate that GCDH inhibits HCC metastasis.

### GCDH modulates DNA repair and autophagy and suppresses HCC progression in vivo

**Fig. 4.**
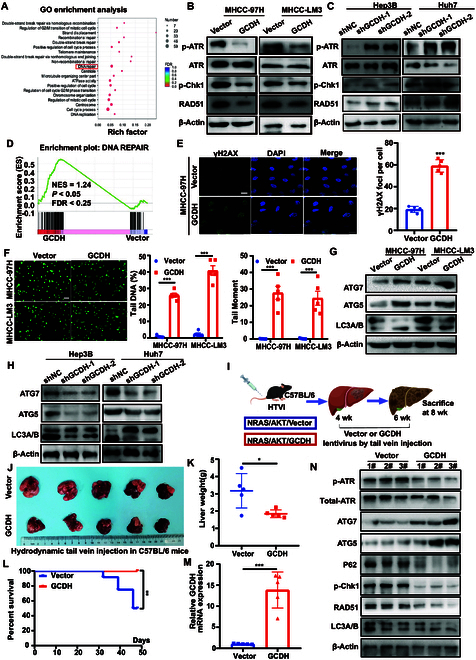
GCDH inhibits DDR and induces autophagy in HCC cells. (A) Ectopic overexpression of GCDH activated the DNA damage signaling pathway according to GO enrichment analysis. (B and C) Expression of p-ATR, ATR, p-Chk1, and RAD51 in GCDH-overexpressing MHCC-97H and MHCC-LM3 cells (B) and GCDH knockdown Hep3B and Huh7 cells (C). (D) GSEA analysis identified DNA repair as top enriched pathways in the GO cohort. (E) γH2AX expression in GCDH-overexpressing MHCC-97H cells was assessed by immunofluorescence. (F) The effect of GCDH overexpression on DNA damage was analyzed by the comet assay. Quantitative results of tail DNA (%) and tail moment are shown on the right. (G and H) Expression of ATG7, ATG5, and LC3A/B in GCDH-overexpressing MHCC-97H and MHCC-LM3 cells (G) and GCDH knockdown Hep3B and Huh7 cells (H). (I to N) In vivo xenograft tumor formation assays were performed by using an SB transposon hydrodynamic transfection system. (I) Schematic of the experimental design. The tumor images (J) and liver weights (K) are shown. (L) The percent survival curves were measured and plotted. (M) GCDH mRNA expression was analyzed in tumor tissues. (N) Immunoblot analysis was performed to examine the expression of DDR and autophagy markers in tumor tissues. * *P* < 0.05; ** *P* < 0.01; *** *P* < 0.001.

To explore the molecular mechanisms underlying GCDH-mediated suppression of HCC progression, RNA sequencing was performed in GCDH-overexpressing HCC cells. Gene Ontology (GO) enrichment analysis revealed that GCDH potentially modulates the DNA repair pathway in HCC cells (Fig. 4A). Due to deficiencies in the DNA damage repair (DDR) mechanisms observed in most tumors, there is an enhanced dependence on ATR pathway signaling when reacting to DNA damage, which includes DNA double-strand breaks (DSBs) [18]. Therefore, we aimed to determine whether GCDH regulates the ATR signaling pathway. Our results showed that overexpression of GCDH decreased the phosphorylation of ATR (p-Ser^428^) and CHK1 (p-Ser^345^), key markers of ATR pathway activation, and led to a marked decrease in RAD51 expression in HCC cells. Conversely, GCDH knockdown had the opposite effect (Fig. 4B and C), suggesting that GCDH negatively regulates the ATR-mediated DDR in HCC. Gene Set Enrichment Analysis (GSEA) revealed marked enrichment of DNA repair-related gene signatures in the high GCDH expression group (normalized enrichment score = 1.24, *P* < 0.05; Fig. 4D). Consistently, we observed a markedly higher level of γH2AX signal and an increased number of γH2AX foci in GCDH-overexpressing cells (Fig. 4E), indicating impaired DDR activation and persistent DNA damage due to reduced repair efficiency. The comet assay, a widely used technique for evaluating DNA damage and repair capacity, revealed that GCDH overexpression markedly increased basal DNA strand breaks in both MHCC-97H and MHCC-LM3 HCC cell lines. Notably, the proportion of DNA present in the tail, an indicator of DNA fragmentation, was significantly elevated in GCDH-overexpressing cells compared to controls (Fig. 4F). These findings suggest that GCDH overexpression impairs the activation of the ATR–CHK1 signaling pathway, leading to the accumulation of unrepaired DNA damage.

Different forms of DNA damage can induce autophagy, while a deficiency in autophagy can result in genomic instability [19]. Recent studies have offered initial insights into the pathways that connect autophagy with the cellular response to DNA damage [20]. Therefore, we next evaluated the effect of GCDH on autophagy. ATG5 plays a central role in autophagosome formation through its participation in the ATG12–ATG5–ATG16L1 complex [21]. We found that overexpression of GCDH up-regulated the expression of ATG5 and ATG7, reduced LC3A protein levels, and induced LC3B expression. Conversely, GCDH knockdown had the opposite effect (Fig. 4G and H), suggesting that GCDH modulates autophagy in HCC cells.

To assess the in vivo tumor-suppressive effects of GCDH during hepatocarcinogenesis, we utilized a well-characterized Sleeping Beauty (SB) transposon-based hydrodynamic injection model [22]. Co-delivery of oncogenic AKT and NRAS with either a control vector or GCDH expression plasmid into the livers of C57BL/6 mice led to full tumor penetrance in both groups. GCDH overexpression markedly reduced tumor burden (Fig. 4J) and tumor weight (Fig. 4K) and prolonged survival compared to the control group (Fig. 4L), demonstrating its inhibitory effect on HCC development. GCDH overexpression in mouse liver tumors was confirmed by quantitative real-time polymerase chain reaction (qRT-PCR) analysis (Fig. 4M). Notably, tumors from the GCDH-overexpressing group showed reduced levels of DDR markers (p-ATR, p-Chk1, and RAD51), along with increased expression of autophagy-related proteins (Fig. 4N), further supporting the role of GCDH in modulating both DDR and autophagy in HCC. Additionally, similar trends were observed in mouse xenograft tumor tissues (Fig. S4).

### GCDH induces cellular ROS accumulation and exacerbates oxidative stress

**Fig. 5.**
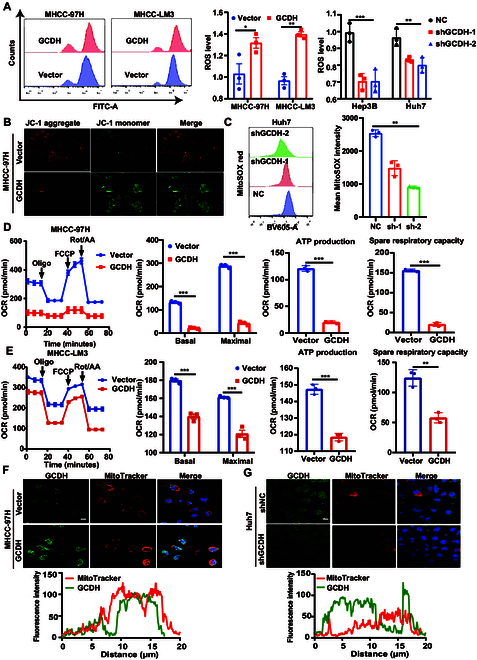
GCDH inhibits OXPHOS and triggers ROS production in HCC cells. (A) The proportion of ROS level in HCC cells with GCDH overexpression or knockdown was analyzed by flow cytometry. Histogram shows the quantification of ROS level. (B) mtMP was determined by JC-1 staining. (C) Mitochondrial ROS were detected by staining with MitoSOX red in Huh7 cells with GCDH knockdown. Bar graph shows quantified MitoSOX fluorescence intensity. (D and E) The effect of GCDH overexpression on OXPHOS was measured with a Seahorse XF96 Analyzer. The basal mitochondrial respiration rate, maximal respiratory capacity, ATP production, and spare respiratory capacity were calculated and statistically analyzed. (F and G) Confocal microscopy with MitoTracker Red and GCDH antibody showed colocalization of GCDH with mitochondria. Hoechst 33342 was used for nuclear staining. Fluorescence intensities were quantified. Scale bars, 10 μm. * *P* < 0.05; ** *P* < 0.01; *** *P* < 0.001.

As part of the acyl-CoA dehydrogenase family of mitochondrial flavoproteins, GCDH catalyzes the oxidative decarboxylation process from glutaryl-CoA to crotonyl-CoA [23]. Previous studies have also shown that GCDH predominantly colocalizes alongside the mitochondrial marker cyclooxygenase IV (COX IV) [4]. Therefore, we investigated the effects of GCDH on the intracellular reactive oxygen species (ROS) levels in HCC. The intracellular ROS levels were elevated by GCDH overexpression, whereas GCDH knockdown had the opposite effects (Fig. 5A and Fig. S5A). Given that mitochondrial membrane potential (mtMP) is a critical indicator of mitochondrial function, we assessed mtMP in GCDH-overexpressing HCC cells using a JC-1 assay. Our results showed that GCDH overexpression significantly reduced mtMP compared to control cells (Fig. 5B and Fig. S5B). Since mitochondria are a major source of intracellular ROS, we next evaluated mitochondrial superoxide levels using MitoSOX staining. Notably, GCDH knockdown cells exhibited markedly lower mitochondrial superoxide production compared to control cells (Fig. 5C).

Given that oxidative phosphorylation (OXPHOS) is a key contributor to ROS production, we assessed the rate of mitochondrial respiration, adenosine triphosphate (ATP) production, and maximal respiratory capacity in control and GCDH-overexpressing cells, respectively. Our results revealed that GCDH overexpression reduced basal cellular respiration, intracellular ATP levels, spare respiratory capacity, and mitochondrial oxygen consumption rate (OCR) (Fig. 5D and E). Furthermore, GCDH overexpression in MHCC-97H cells markedly enhanced the colocalization of GCDH with MitoTracker, a mitochondrial marker, whereas GCDH knockdown in Huh7 cells reduced this overlap (Fig. 5F and G). These findings indicate that GCDH is predominantly localized to mitochondria in HCC cells and support its potential involvement in regulating mitochondrial function and oxidative stress.

### Inhibition of ROS or autophagy abolished GCDH-induced DNA damage and autophagy

**Fig. 6.**
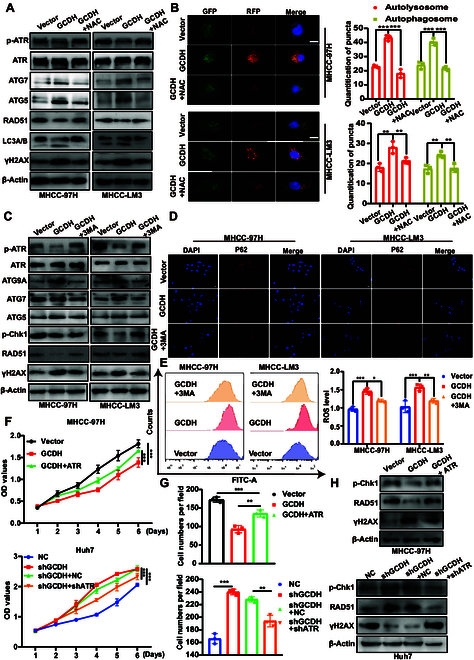
Inhibition of ROS and autophagy abolished the effects of GCDH on DNA damage and autophagy. (A) WB analysis showed the expression of DDR markers and autophagy-related markers in GCDH-overexpressing MHCC-97H and MHCC-LM3 cells treated with or without NAC (5 mM). (B) Representative images of GCDH overexpression cells treated with NAC after transfecting with mRFP-GFP-LC3. Hoechst 33342 was used for nuclear staining. (C) WB analysis showed the expression of DDR markers and autophagy-related markers in GCDH overexpression HCC cells treated with or without 3-MA (10 mM for 2 h). (D) GCDH-overexpressing HCC cells incubated with 3-MA for the indicated time were subjected to an immunofluorescence assay. (E) ROS levels in HCC cells transfected with vector or GCDH, with or without 3-MA pretreatment (10 mM), were measured by H_2_DCFDA staining and flow cytometry. (F and G) GCDH-overexpressing and knockdown HCC cells were transfected with ATR or shATR, respectively, and cell proliferation was evaluated by CCK-8 assay (F) and colony formation assay (G). (H) GCDH-overexpressing and knockdown HCC cells were transfected with ATR or shATR, respectively, and DDR markers were detected by WB. Scale bars, 10 μm. * *P* < 0.05; ** *P* < 0.01; *** *P* < 0.001.

Given that ROS might have a marked impact on suppressing cancer cell metastasis through GCDH-mediated autophagy and DNA damage, we investigated whether the ROS inhibitor *N*-acetyl-l-cysteine (NAC) could modulate these downstream effects. The NAC treatment substantially enhanced the levels of phosphorylated ATR (p-Ser^428^) and RAD51 and decreased the expression of γH2AX. NAC treatment successfully inhibited the increased expression of autophagy markers (ATG7 and ATG5) induced by GCDH overexpression (Fig. 6A). Understanding the status of autophagic flux is essential for elucidating the functions of autophagy and identifying related pathological conditions. Notably, NAC treatment effectively reversed the accumulation of autophagosomes and autolysosomes induced by GCDH overexpression in HCC cells (Fig. 6B).

To elucidate the relationship between GCDH-mediated autophagy and DNA damage, overexpressing GCDH HCC cells were treated with the autophagy inhibitor 3-methyladenine (3-MA). 3-MA suppressed the up-regulation of ATG9A, ATG7, and ATG5 in GCDH-overexpressing cells. Furthermore, this inhibition was accompanied by increased phosphorylation of ATR and Chk1, elevated RAD51 expression, and reduced γH2AX levels (Fig. 6C). In addition, we found that 3-MA attenuated ROS production induced by GCDH overexpression (Fig. 6E). Overall, these results demonstrate that GCDH induces DNA damage and promotes autophagosome formation, ultimately leading to autophagy.

To further explore the role of ATR on GCDH-related HCC proliferation and DNA damage, we examined the impact of ATR on cell proliferation and DNA damage responses in HCC cells. Our results demonstrated that ATR overexpression partially rescued the growth suppression caused by GCDH overexpression, whereas ATR knockdown counteracted the proliferative advantage conferred by GCDH knockdown (Fig. 6F and G and Fig. S6). Furthermore, ATR overexpression enhanced the phosphorylation of Chk1 and expression of RAD51 while decreasing γH2AX levels, indicative of enhanced DNA repair. In contrast, ATR knockdown abrogated the up-regulation of p-Chk1 and RAD51 induced by GCDH silencing (Fig. 6H), highlighting ATR as a key mediator of GCDH-dependent DNA damage signaling in HCC.

### GCDH-K438 acetylation is critical for the tumor-suppressive function of HCC cells

**Fig. 7.**
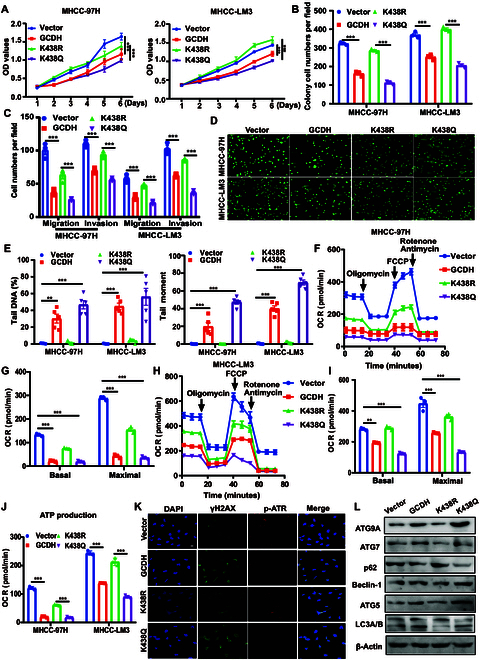
GCDH-K438 acetylation controls its tumor-suppressive role in HCC cells. (A and B) CCK-8 (A) and colony formation (B) assays show the effect of GCDH K438 acetylation on cell proliferation. (C) Transwell assays assess cell migration and invasion. (D and E) Comet assay evaluates DNA damage (D), with quantitative analysis of tail DNA (%) and tail moment (E). (F to J) Mitochondrial OXPHOS was measured using a Seahorse XF96 Analyzer (F and H). Mitochondrial function (basal respiration, maximal respiratory capacity, ATP production) was calculated and statistically analyzed (G, I, and J). (K) γH2AX and p-ATR expression was detected by immunofluorescence. (L) Autophagy-related proteins were analyzed by WB. ** *P* < 0.01; *** *P* < 0.001.

To evaluate the role of GCDH acetylation in the progression of HCC, we established stable cell lines expressing GCDH-WT, GCDH-K438R, or GCDH-K438Q. Functional assays revealed that GCDH-K438Q markedly suppressed cell proliferation, colony formation, migration, and invasion, whereas GCDH-K438R had the opposite effect, compared to GCDH-WT (Fig. 7A to C and Fig. S7A and B). In addition, we found that GCDH-K438Q increased cleaved caspase-3 expression, whereas GCDH-K438R decreased cleaved caspase-3 expression in HCC cells (Fig. S8). In the in vivo mouse xenograft model, GCDH-K438Q mutant decreased HCC tumor growth, whereas GCDH-K438R mutant increased HCC tumor growth (Fig. S9).

We next determined the effects of GCDH-K438 acetylation on the DNA damage, OXPHOS, and autophagy. Comet assays showed a marked increase in DNA tail intensity in cells expressing GCDH-K438Q, indicating elevated DNA damage, whereas GCDH-K438R exhibited reduced DNA damage compared to GCDH-WT (Fig. 7D and E). The seahorse results revealed that GCDH-K438Q mutant reduced the mitochondrial OCR and the intracellular ATP levels, reflecting impaired mitochondrial respiration and OXPHOS, whereas the GCDH-K438R mutant had the opposite effect (Fig. 7F to J). These results suggest that acetylation at K438 modulates cellular energy metabolism. Immunofluorescence staining demonstrated that the GCDH-K438Q mutant markedly increased γH2AX expression while reducing phosphorylated ATR levels (Fig. 7K). Conversely, the GCDH-K438R mutant showed reduced γH2AX and elevated p-ATR expression. Additionally, autophagy activity was markedly increased in GCDH-K438Q-expressing cells but decreased in GCDH-K438R-expressing cells (Fig. 7L), further supporting the role of K438 acetylation in modulating DNA damage and autophagy.

### GCDH down-regulation is correlated with unfavorable prognosis in HCC patients

**Fig. 8.**
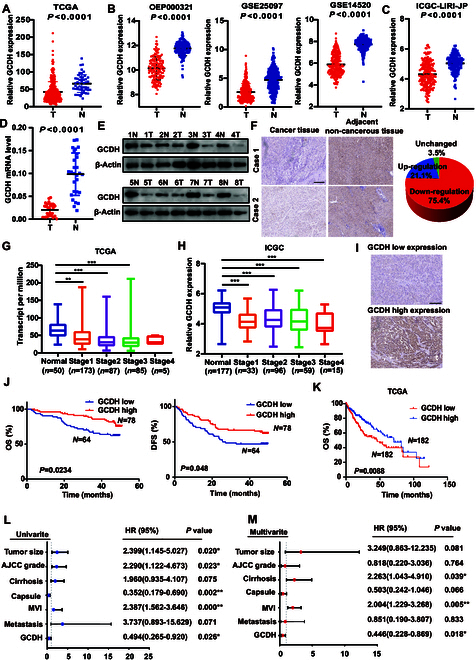
GCDH down-regulation is associated with poor prognosis in HCC. (A to C) GCDH expression in HCC tissues was compared with that in the corresponding noncancerous liver tissues in the TCGA dataset (*n* = 50) (A), OEP000321, GSE25097, and GSE14520 (B), and ICGC-LIRI-JP cohort (C). (D and E) qPCR (D) and WB (E) were performed to compare GCDH mRNA and protein expression levels in HCC tissues and adjacent noncancerous tissues. (F) Representative IHC images showing GCDH expression in HCC tissues and matched noncancerous liver tissues. (G and H) GCDH expression in HCC tissues of different grades was analyzed in TCGA (G) and ICGC-LIRI-JP (H) cohorts. (I) Representative IHC images of HCC tissues exhibiting high and low GCDH expression. (J) OS and DFS of HCC patients stratified by the GCDH expression level. (K) OS analysis of HCC patients in the TCGA cohort. (L and M) Univariate and multivariate Cox proportional hazards analyses were conducted to evaluate the hazard ratio (HR) of GCDH in terms of the OS of patients with HCC. *P < 0.05; **P < 0.01; ***P < 0.001.

Transcriptomic profiling across multiple public databases, including The Cancer Genome Atlas (TCGA), Gene Expression Omnibus (GEO), and JP Project of the International Cancer Genome Consortium (ICGC-LIRI-JP), revealed the down-regulation of GCDH in HCC tissues compared to nontumor tissues (Fig. 8A to C). This observation was corroborated at both mRNA and protein levels using qPCR and Western blotting (WB) (Fig. 8D and E), and further validated by immunohistochemistry (IHC) staining (Fig. 8F). In addition, analysis of TCGA data revealed down-regulation of GCDH in patients across various tumor types (Fig. S10A). We further investigated the clinical relevance of GCDH in HCC patients and found that its expression was inversely correlated with tumor grade in both the TCGA and ICGC-LIRI-JP cohorts (Fig. 8G and H). Based on the IHC results, patients were stratified into 2 groups according to their GCDH expression levels (Fig. 8I). Statistical analysis revealed a marked negative correlation between GCDH expression and serum α-fetoprotein (AFP) levels, as well as an association with the absence of tumor capsule formation (Table S5).

We further evaluated the prognostic significance of GCDH expression in HCC patients. Kaplan–Meier survival analysis demonstrated that patients with low GCDH expression had markedly worse overall survival (OS; *P* = 0.0234) and disease-free survival (DFS; *P* = 0.048) compared to those with high GCDH expression (Fig. 8J). These findings were consistently validated using data from TCGA, GEO, and ICGC databases (Fig. 8K and Fig. S10B). Moreover, univariate and multivariate Cox proportional hazard analyses indicated that low GCDH expression was an independent predictor of worse survival outcomes in HCC patients compared to those with high GCDH expression (Fig. 8L and M). In addition, high expression of GCDH was associated with improved OS in patients with various types of tumors according to TCGA data (Fig. S11). Therefore, these findings indicate that the absence of GCDH is correlated with worse prognosis in HCC patients.

## Discussion

PTMs, including phosphorylation, ubiquitination, acetylation, and methylation, are critical regulators of cellular processes such as proteostasis and metabolic homeostasis. Among all types of PTMs, acetylation is a particularly common modification, affecting thousands of proteins, especially metabolic enzymes [24]. Research has shown that dysregulation of histone modifications can lead to aberrant transcriptional programs, which in turn enhance the initiation and advancement of HCC [25]. Although the impact of GCDH on lysine metabolism and protein glutarylation has been extensively studied in various tumor types, the regulatory role of GCDH acetylation remains largely unexplored in HCC. In this study, we revealed that GCDH acetylation is dynamically controlled by the acetyltransferase P300 and the deacetylase HDAC1, identifying a novel regulatory axis in GCDH function. In addition to histones, P300 acetylates a range of nonhistone proteins, including other epigenetic enzymes and transcription factors. As a key acetyltransferase, P300 targets proteins that play critical roles in essential biological processes such as cell proliferation, apoptosis, differentiation, and the DNA damage response [2,26]. P300 promotes HCC progression by facilitating the acetylation of specific oncogenes and boosting their transcription [27,28]. We detected an elevation in the acetylation levels of GCDH following P300 overexpression. In addition to its well-characterized histone acetyltransferase (HAT) activity, P300 has been shown to catalyze histone lysine crotonylation (HCT activity) [29]. Previous research showed that GCDH inhibited the progression of HCC by inducing crotonylation, which suppressed the pentose phosphate pathway (PPP) and glycolysis, ultimately triggering the senescence process in HCC cells [6]. It has been demonstrated that high expression of histone deacetylases HDAC1 and HDAC3 is affiliated with poorer prognosis in HCC patients [30,31]. Our study demonstrated that HDAC1-mediated GCDH deacetylation led to GCDH inactivation, which promoted HCC progression. In addition, increased crotonylation level through the knockdown of HDACs or the addition of the HDAC inhibitor TSA inhibited HCC progression [32]. Therefore, P300 acts as a promiscuous acyltransferase capable of catalyzing both acetylation and crotonylation of GCDH, while HDAC1 mediates deacetylation and potentially decrotonylation of the same modifications in HCC.

Emerging evidence indicates that posttranslational acetylation of proteins plays a key regulatory role in various metabolic pathways by influencing enzymatic activity, structural stability, cellular compartmentalization, and protein–protein interactions [33]. Nonetheless, several studies suggests that global changes in protein acetylation levels do not always correlate strongly with alterations in enzymatic activity [34]. As GCDH activity and its associated metabolic changes were not assessed in the current study, we are unable to determine whether acetylation directly influences GCDH enzyme activity. Further investigation will be conducted in future work.

 Lysine and tryptophan are metabolized through a common metabolic pathway catalyzed by the dehydrogenase DHTKD1. In this pathway, DHTKD1 catalyzes the synthesis of the intermediate product glutaryl-CoA. GCDH catalyzes the conversion of glutaryl-CoA to crotonyl-CoA, which is subsequently metabolized to acetyl-CoA for entry into the TCA cycle [35,36]. GCDH inhibition results in elevated glutarate levels and promotes protein glutarylation independent of lysine catabolism [37]. In melanoma cells, the loss of GCDH triggers cell death programs that can be prevented by inhibiting DHTKD1. These findings reveal that GCDH is a key metabolic regulator of lysine catabolism and is essential for maintaining cell viability [4]. Moreover, in GSCs, reprogramming of lysine catabolism is characterized by up-regulation of SLC7A2 and GCDH, along with the down-regulation of crotonyl-CoA hydratase ECHS1. This metabolic shift results in increased intracellular crotonyl-CoA levels and enhanced lysine crotonylation of histone H4 [7]. However, our results revealed that GCDH acetylation exerts tumor-suppressive effects on HCC proliferation and metastasis, independently of the regulation of other key enzymes in lysine metabolism (Fig. S12).

Autophagy functions as a quality control system that maintains cellular homeostasis through the recycling of surplus and dysfunctional organelles [38]. Various types of DNA damage have the potential to provoke autophagy, and autophagy deficiency leads to genomic instability [39]. A comprehensive analysis of autophagy targets could uncover the physiological role of autophagy induced by DNA damage. Autophagy exhibits dual roles in carcinogenesis and cancer progression, acting both as a tumor suppressor and, in certain contexts, as an oncogene [40–42]. Autophagy suppresses tumorigenesis and tumor progression by clearing away damaged proteins and organelles to safeguard against genomic damage. Consistent with this, our results showed that GCDH overexpression promotes autophagy and inhibits tumor initiation and development by activating oxidative stress, leading to DNA damage and impaired DNA repair.

Recent studies have shown that cells deficient in poly(adenosine diphosphate–ribose) polymerase 1 (PARP1) and autophagy exhibit increased sensitivity to DNA damage caused by elevated ROS levels derived from dysfunctional mitochondria. It has therefore been proposed that autophagy is fundamental to promoting cell survival after DNA damage [43]. Nucleophagy mediated by TEX264 contributes to the stability of DNA replication forks, ensures genetic stability, and facilitates cell survival, highlighting its potential clinical relevance for patients with colorectal cancer [44]. Autophagy induced by DNA damage can selectively degrade distinct proteins, including Rnr1, USP14, CHK1, and NOXA, thereby directly or indirectly impacting DNA repair and determining cell fate regarding proliferation, apoptosis, or senescence. Upon encountering DNA damage, cells stimulate the DDR responses, which help recognize the damaged DNA and recruit DNA repair proteins at the damage sites to aid in repair [45]. If the damage repair pathways are disrupted, it may result in mutations or inactivation of specific oncogenes or tumor suppressor genes, leading to either the death of normal cells or their conversion into tumor cells [45].

The accumulation of replication protein A (RPA)-coated single-stranded DNA (ssDNA) resulting from DNA damage and replication fork stalling activates ATR kinase, which subsequently phosphorylates CHK1. This phosphorylation temporarily pauses cell cycle progression, enabling the necessary DNA repair processes [46]. Notably, numerous standard chemotherapies employed in cancer treatment achieve their therapeutic properties by inducing DNA damage. There is ongoing clinical investigation into the combination of ATR inhibitors with agents that disrupt DNA replication [47]. In addition to inhibiting DNA repair, ATR inhibitors induce unscheduled firing of replication origins, which can result in cancer cell death through genomic alterations caused by faulty replication and subsequent mitotic catastrophe [48,49]. In this study, ATR knockdown effectively reversed the elevated expression of DDR markers (p-Chk1 and RAD51) induced by GCDH knockdown. While ATR signaling typically leads to a transient replication arrest that allows cells to repair damage and resume replication [50], GCDH appears to induce a more permanent suppression of replication and cellular growth. Overexpression of GCDH induced DNA damage and impaired DNA repair by suppressing the ATR/Chk1 checkpoint pathway, leading to the accumulation of damaged DNA and subsequent cell death. Therefore, activation of the ATR-dependent checkpoint pathway was impaired in GCDH overexpression cells.

**Fig. 9.**
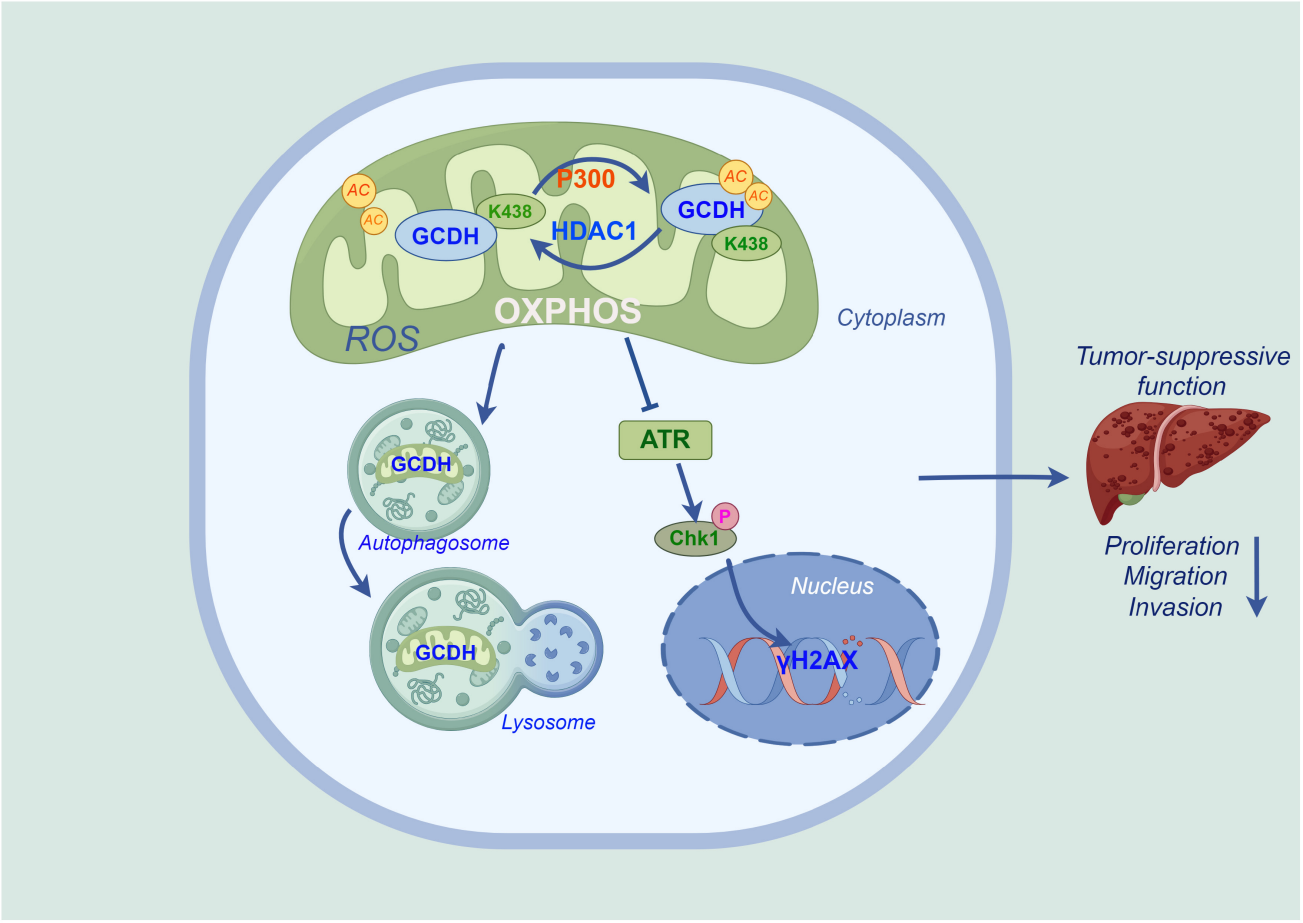
Schematic diagram illustrating the mechanism by which GCDH down-regulation promotes HCC progression. GCDH is localized to the mitochondria and undergoes acetylation at residue K438 by the acetyltransferase P300 and deacetylase HDAC1 in HCC cells. GCDH inhibits the ATR–CHK1 signaling axis and promotes ROS-mediated activation of autophagy, leading to the inhibition of HCC proliferation and metastasis.

Overall, our findings revealed that GCDH K438 acetylation inhibits HCC progression through inhibition of the ATR–CHK1 axis and ROS-mediated activation of autophagy, highlighting its potential as a therapeutic strategy for preventing HCC progression (Fig. 9).

## Materials and Methods

### Cell lines and cell culture

Huh7 and Hep3B cells were acquired from Riken Cell Bank (Tsukuba, Japan). We procured HEK293T cell lines from the American Type Culture Collection (Manassas, VA, USA). The MHCC-LM3 and MHCC-97H cell lines were sourced from the Liver Cancer Institute, Zhongshan Hospital of Fudan University (Shanghai, China). The HCC cell lines employed in this research were grown in Dulbecco’s modified Eagle’s medium (DMEM) (Gibco) supplemented with 10% heat-inactivated fetal bovine serum (FBS) (Gibco). Cells were cultured in a 37 °C incubator with 5% CO_2_ and high humidity. The respective suppliers authenticated and characterized all cell lines. To maintain cellular integrity, the cell lines were thawed from verified stocks and employed within 4 passages. Consistent quality checks on morphology and growth patterns were conducted to ensure mycoplasma-free status and phenotypic stability of the cell lines.

### Lentivirus production and cell transduction

The GCDH lentiviral overexpression plasmid and shRNA plasmid were obtained from the CCSB-Broad Lentiviral Expression Library and Human TRC shRNA Library. The ATR plasmid was maintained in our laboratory, and its sequence was confirmed by sequencing from the 5′ and 3′ ends. The target sequences can be found in Table S1.

To generate viral particles, the GCDH overexpression or shRNA plasmid, the packaging plasmid psPAX2, and the envelope plasmid pMD2.G (Addgene) were cotransfected into HEK293T cells utilizing Lipofectamine 2000 (Invitrogen). After 72 h of transfection, harvesting of the viruses was performed, followed by titer determination. The treatment of HCC cells involved 1 × 10^6^ recombinant lentivirus-transducing units and the addition 6 μg/ml polybrene (Sigma).

### Quantitative real-time PCR

Total RNA was extracted, followed by reverse transcription and qRT-PCR analyses, in accordance with previously established protocols [51]. An ABI Prism 7500 System (Applied Biosystems, Carlsbad, CA, USA) was used in combination with SYBR Premix Ex Taq (Takara, Dalian, China) for qRT-PCR. The levels of mRNA expression were standardized to the housekeeping gene glyceraldehyde-3-phosphate dehydrogenase (GAPDH). The primer sequences utilized in the experiments can be available in Table S2.

### Co-immunoprecipitation and WB analysis

For immunoprecipitation, protein lysates were combined with the specific antibodies on an orbital shaker overnight at 4 °C, followed by supplementing 50 μl of protein A/G agarose beads. Following that, the beads were washed 3 times using lysis buffer to remove any nonspecific binding. After washing, the protein solution or the beads were subjected to boiling in sodium dodecyl sulfate–polyacrylamide gel electrophoresis (SDS-PAGE) loading buffer for 10 min at 100 °C to denature the proteins and analyzed by WB.

For WB analysis, total cell proteins were separated by SDS-PAGE and transferred onto polyvinylidene difluoride membranes (Millipore). Membranes were blocked and incubated with primary antibodies overnight at 4 °C. After washing, membranes were incubated with horseradish peroxidase (HRP)-conjugated secondary antibodies for 1 h at room temperature. Immunoreactive bands were visualized using an enhanced chemiluminescence (ECL) detection reagent (Pierce, Rockford, IL, USA). β-Actin was utilized as a loading control to ensure equal protein loading. Information regarding the antibodies employed can be found in Table S3.

### GST pull-down assay

Fusion proteins GST, GST-P300, and GST-HDAC1 were obtained from Proteintech Group (Proteintech, Wuhan, China). The GST pull-down assay was carried out as previously described [52]. In brief, 50 μl of Glutathione–Sepharose 4B beads (GE Healthcare), coupled with GST or GST-P300/HDAC1 fusion protein, was incubated with GCDH overexpression MHCC-97H whole-cell lysates for 2 h at 4 °C. Afterward, following the removal of the supernatant, 3 repetitions of washing the beads with the reaction buffer were carried out. The bound proteins were eluted by heating the samples in SDS-PAGE loading buffer and analyzed by WB using the appropriate antibodies.

### Cell proliferation, colony formation assays, and EdU assay

Cell proliferation was evaluated following the manufacturer’s instructions using Cell Counting Kit-8 (CCK-8; Bimake, USA). The absorbance of the wells was determined at 450 nm using a spectrophotometer.

Colony formation assays were conducted following previously described protocols [46]. Cells were seeded in 6-well plates and maintained at 37 °C with 5% CO_2_ in a humidified environment for 2 weeks. After being fixed with 4% phosphate-buffered formalin (pH 7.4) for 30 min, the resulting colonies were stained with Giemsa.

EdU staining was performed using the Cell Proliferation EdU Image Kit from Abbkine (Wuhan, China). Cells were processed according to the manufacturer’s instructions. Following EdU staining, the cells were visualized and captured using a fluorescence microscope.

To confirm the reliability and reproducibility of the findings, all experiments were conducted 3 times.

### Migration and invasion assays

Cells were seeded in serum-free medium in the upper chamber of uncoated (for migration) or Matrigel-coated (for invasion) Transwell inserts (8-μm pore, BD Biosciences). The lower chamber contained DMEM with 10% FBS as a chemoattractant. After 24 or 48 h, nonmigrated or noninvaded cells were removed with a cotton swab. The migrated or invaded cells were fixed with 4% paraformaldehyde at room temperature for 30 min and subsequently stained with crystal violet solution. Using a microscope, the count of cells was conducted in 5 randomly selected fields of view.

### Flow cytometry analysis

For cell cycle analysis, cells underwent 2 washes with cold phosphate-buffered saline (PBS) before being fixed in 70% cold ethanol, followed by overnight incubation. Prior to analysis, cells were treated with a staining solution comprising 10 mg/ml ribonuclease and 400 mg/ml propidium iodide (PI) and then incubated for 30 min at 37 °C. Subsequently, the cells were analyzed using flow cytometry to determine their cell cycle distribution.

For intracellular ROS analysis, cells were inoculated into 6-well plates and incubated overnight. The cells were treated with 2 μM chloromethyl-20,70-dichlorodihydrofluorescein diacetate (CM-H_2_DCFDA, Life Technologies). After a 30-min incubation at 37 °C, cells were analyzed by flow cytometry. Data were processed using FlowJo software.

For mitochondrial ROS analysis, HCC cells were incubated with 2.5 μM MitoSOX Red mitochondrial superoxide indicator (Invitrogen, Carlsbad, CA) for 30 min at 37 °C in the dark. Cells were then washed and resuspended in fluorescence-activated cell sorting buffer before analysis by flow cytometry.

### Measurement of mtMP

To evaluate mtMP, cells were incubated with 10 μM JC-1 dye (Yeasen, Shanghai, China) at 37 °C for 30 min. After washing with JC-1 staining buffer, cells were examined under a fluorescence microscope. In healthy mitochondria with high membrane potential, JC-1 forms aggregates that emit red fluorescence. In contrast, in depolarized or unhealthy mitochondria, JC-1 exists as monomers that produce green fluorescence. Nuclei were counterstained with Hoechst 33342.

### Measurement of mitochondrial oxygen consumption capacity

The OCR was analyzed using the Seahorse Bioscience XF96 Extracellular Flux Analyzer (Seahorse Bioscience, USA) in combination with the Seahorse XF Cell Mito Stress Test Kit (Agilent) [53]. Briefly, HCC cells (2 × 10^4^/well) were plated into an XF96-well plate and allowed to incubate overnight. The next day, an unbuffered medium was used to incubate the cells, followed by a series of injections including 1.5 μM oligomycin, 2.0 μM carbonyl cyanide *p*-trifluoromethoxyphenylhydrazone (FCCP), and 0.5 μM Rot/AA to determine OCR. Ultimately, the OCR values were adjusted based on the protein amounts of the cells prior to group comparisons.

### Immunofluorescent confocal imaging

HCC cells were seeded on Lab-Tek chamber slides (Nunc) and allowed to attach for 24 h. Cells were fixed with 4% paraformaldehyde in PBS for 30 min, permeabilized with 0.1% Triton X-100 in PBS for 5 min, and blocked before incubation with primary antibodies. Fluorescent detection was performed using Alexa Fluor 488- or Alexa Fluor 555-conjugated secondary antibodies for 90 min at 37 °C. 4′,6-Diamidino-2-phenylindole (DAPI) served as the staining agent for nuclei. Images were captured using a Leica TCS SP8 confocal microscope (Leica Microsystems). Details of the antibodies are listed in Table S3.

### Autophagic flux analysis

HCC cells received mCherry-GFP-LC3 lentiviral vectors (GeneChem, Shanghai, China) by transfection following the guidelines provided by the manufacturer. After puromycin selection for 2 weeks, confocal fluorescence microscopy (Leica, Microsystems) was used to capture images. The quantification of yellow or red fluorescence was performed to monitor the progression of autophagic flux. The count of puncta per cell was conducted using 5 random images for further analysis.

### Alkaline comet assay

The comet assay was performed using Trevigen’s Comet Assay kit (Trevigen, Gaithersburg, MD) according to the manufacturer’s instructions. Briefly, cells were harvested and suspended in low-melting-point agarose, which was then applied to CometSlides for subsequent electrophoresis and DNA damage assessment. The minigels were then dehydrated by incubating Gelbond films first in 70% ethanol for 15 min and then in 100% ethanol for 30 min. Following cell lysis, the remaining nucleoids were subjected to electrophoresis. DNA damage was then visualized by staining with SYBR Gold and examining the samples under a fluorescence microscope for the presence of comet tails. The extent of DNA damage was quantified as tail moment using CASP software.

### In vivo mice model

To evaluate the in vivo growth and metastasis of HCC cells, HCC xenograft orthotopic models were established using 6-week-old male BALB/c nude mice. A total of 2 × 10^6^ HCC cells were suspended in 40 μl of a 1:1 mixture of serum-free DMEM and Matrigel (BD Biosciences), and injected into the left liver lobe through a small abdominal incision using a microsyringe. Mice were monitored for 6 weeks. At the end of the experiment, the mice were euthanized, and the tissues were harvested. Liver and lung tissues were fixed in neutral formalin buffered with phosphate for at least 72 h. Lung metastases were assessed via hematoxylin and eosin (H&E) staining of tissue sections.

To induce liver tumor formation, hydrodynamic transfection (HTVi) was performed using the SB transposon system as previously described [54]. Briefly, 20 μg of pT3-myr-AKT-HA (Addgene, plasmid #31789), 20 μg of pT/Caggs-NRASV12 (Addgene, plasmid #20205), and pCMV-Vector-Lv105 or pCMV-GCDH-Lv105 (Gene Copoeia, Guangzhou) together with 5 μg of pCMV (CAT)T7-SB100 transposase (Addgene, plasmid #34879) were diluted in 2 ml of saline (0.9% NaCl) at a 25:1 DNA-to-transposase ratio. The mixture was rapidly injected via the tail vein into 6- to 8-week-old C57BL/6 mice over 5 to 7 s. After 8 weeks, mice were euthanized and examined for liver tumor development based on palpable abdominal masses and histopathological analysis. All animal experiments were conducted in compliance with applicable laws and received approval from the Renji Hospital Institutional Animal Care and Use Committee (KY2021-203-B), following the guidelines outlined in the Institutional Guide for the Care and Use of Laboratory Animals.

### Immunohistochemistry

IHC assays were performed as previously described [55]. A total of 142 paired human HCC and adjacent noncancerous tissues were sourced from Zhongshan Hospital of Fudan University (Shanghai, China). Table S4 displays the clinicopathological features of HCC patients. All samples were acquired with the participants’ informed consent. This study followed the guidelines and recommendations outlined in the Ethics Committee of Renji Hospital, Shanghai Jiao Tong University School of Medicine (KY2021-203-B). In brief, the sections were deparaffinized and rehydrated in sodium citrate buffer (pH 6.0) to facilitate antigen retrieval, and endogenous peroxidase activity was blocked with 3% hydrogen peroxide, then sequentially incubated overnight at 4 °C with the primary antibody, followed by incubation with an HRP-conjugated secondary antibody, and then incubated with diaminobenzidine (DAB) solution used to stain the nuclei. Protein expression was analyzed using both the intensity and density of immunostaining. The intensity of staining was categorized as negative (0), weak (1), moderate (2), or high (3). The density of positive tumor cells was scored as follows: 0% (0), 1% to 10% (1), 11% to 35% (2), 36% to 75% (3), and >76% (4). The final score was calculated by multiplying these 2 values.

### Statistical analysis

All the data are reported as mean ± SD. For the comparison of means between 2 groups, an unpaired *t* test was executed, while one-way analysis of variance (ANOVA) was used to assess the means of several groups. Using the Kaplan–Meier method, survival curves were plotted and subsequently compared utilizing the log-rank test, with statistical significance defined as *P* < 0.05.

## Ethical Approval

This study followed the guidelines and recommendations outlined in the Ethics Committee of Renji Hospital, Shanghai Jiao Tong University School of Medicine (KY2021-203-B). Informed consent was obtained and accepted by all of the patients before enrolment. All methods were performed according to relevant guidelines and regulations. All animal experiments were approved by the Laboratory Animal Ethics Committee of the Renji Hospital, Shanghai Jiao Tong University School of Medicine (approval number: RT2022-122u). All methods are reported in accordance with ARRIVE guidelines for the reporting of animal experiments.

## Data Availability

The data that support the findings of this study are available from the corresponding author upon reasonable request.
