# Supplementary material for: GCDH Acetylation Orchestrates DNA Damage Response and Autophagy via Mitochondrial ROS to Suppress Hepatocellular Carcinoma Progression
Source: Research (Wash D C). 2025 Aug 29;8:0862. doi: 10.34133/research.0862 (PMC12395558; doi:10.34133/research.0862)
Supplement: Supplementary 1 — Figs. S1 to S12 Tables S1 to S5 [file research.0862.f1.docx]

**GCDH acetylation orchestrates DNA damage response and autophagy via mitochondrial ROS to suppress hepatocellular carcinoma progression**

Wei Tian^1^, Yue Yang^1^, Lili Meng^2^, Chao Ge^1^, Yuqi Liu^1^, Canxue Zhang^1^, Zhihong Huang^1^, Chi Zhang^1^, Hua Tian^1.3.4*^

^1^State Key Laboratory of Systems Medicine for Cancer, Shanghai Cancer Institute, Renji Hospital, Shanghai Jiao Tong University School of Medicine, Shanghai, China

^2^Department of Pathology, Zhongshan Hospital, Fudan University, Shanghai, China

^3^Department of Pathology, The Affiliated Hospital of Youjiang Medical University for Nationalities, Baise 533000, China

^4^The Key Laboratory of Molecular Pathology (Hepatobiliary Diseases) of Guangxi, Baise 533000, China

**^*^Correspondence**：*Hua Tian, Ph.D.,* State Key Laboratory of Systems Medicine for Cancer*, Shanghai Cancer Institute, Renji Hospital, Shanghai Jiao Tong University School of Medicine, 25/Ln 2200, Xietu Road, Shanghai 200032, China. Tel/Fax: +86-21-64432140，E-mail:* [*htian@shsci.org*](mailto:htian@shsci.org)

**
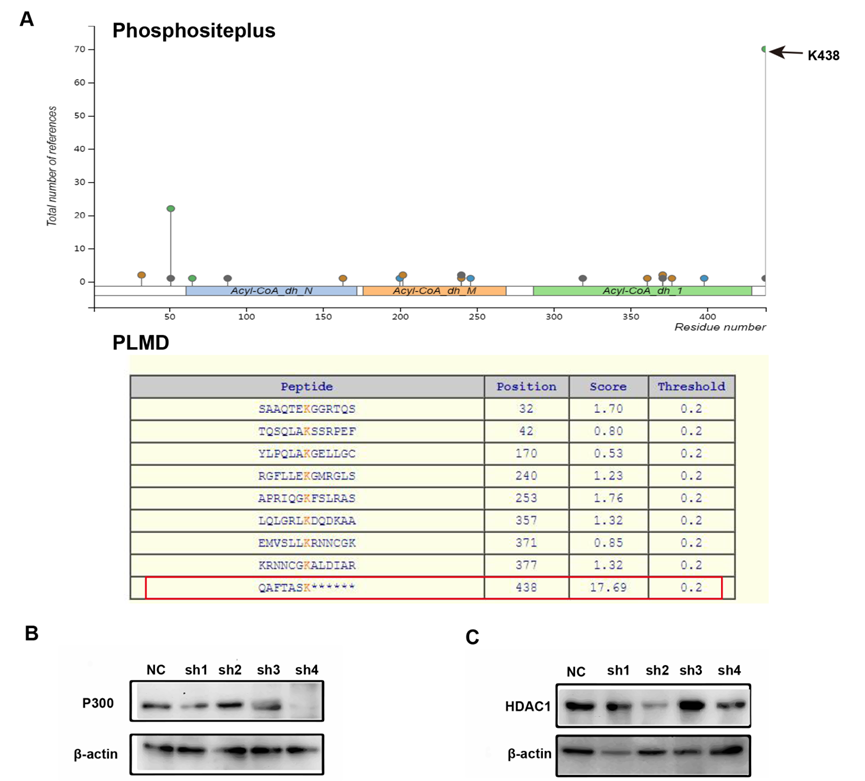
**

**Fig. S1**. (A) The predicted acetylation site of GCDH analyzed by publicly available PhosphoSitePlus and PLMD databases. (B) The expression of P300 knockdown was analyzed by WB in HEK293T cells. (C) The expression of HDAC1 knockdown was analyzed by WB in HEK293T cells.


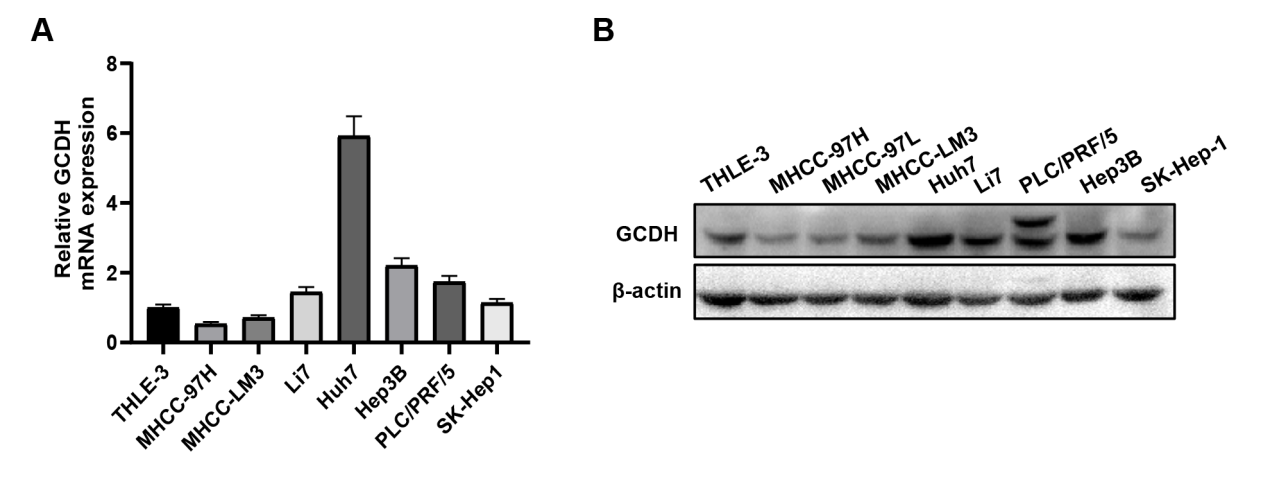


**Fig. S2**. (A)The expression of GCDH was analyzed by qPCR in HCC cell lines. (B) The expression of GCDH was analyzed by WB in HCC cell lines.


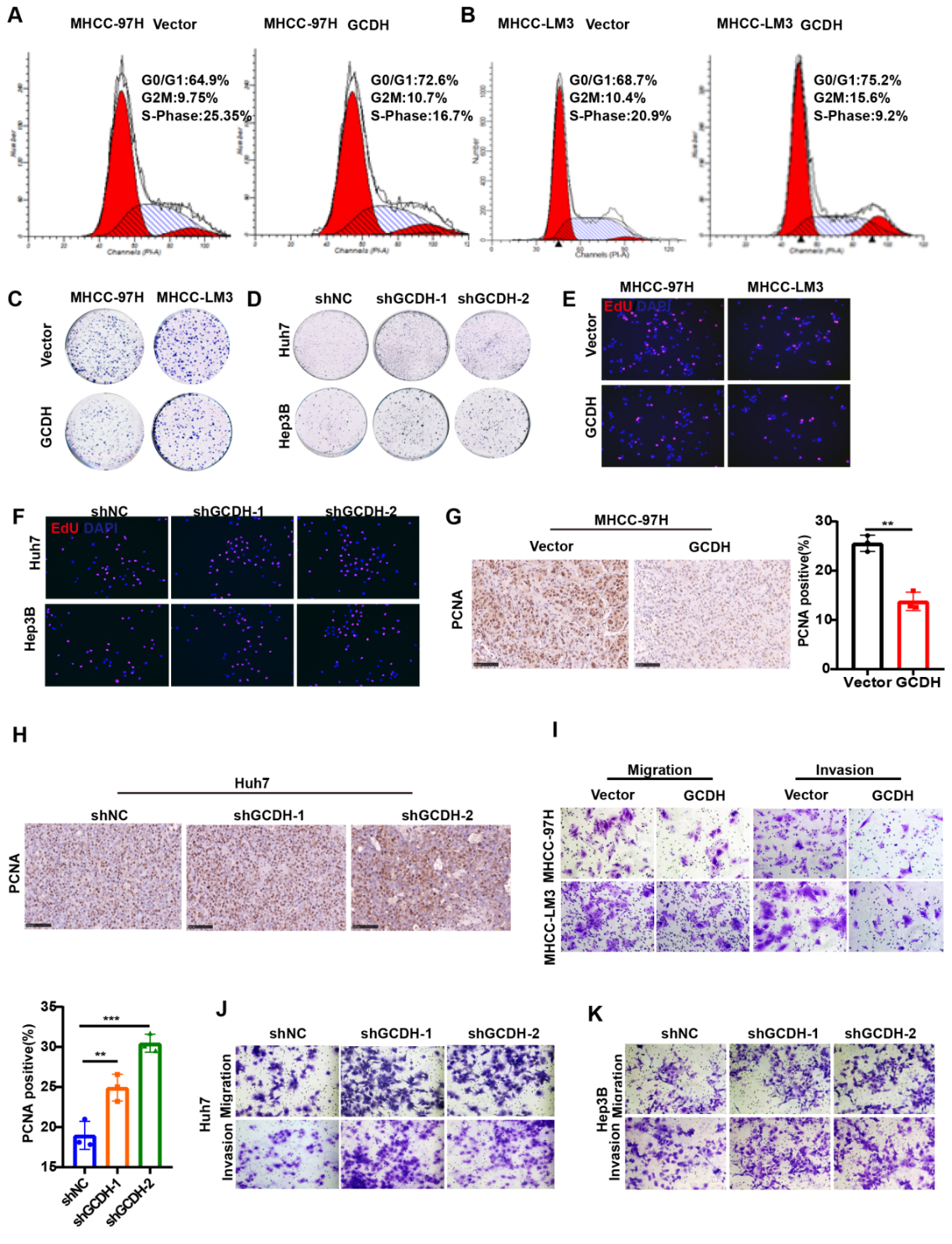


**Fig.S3.** (A) The cell cycle distribution of GCDH overexpression MHCC-97H was analyzed by flow cytometry. (B) The cell cycle distribution of GCDH overexpression MHCC-LM3 was analyzed by flow cytometry. (C)The effect of GCDH overexpression on HCC cell proliferation was assessed by colony formation assays. (D) The effect of GCDH knockdown on HCC cell proliferation was assessed by colony formation assays. (E, F) The effect of GCDH overexpression and knockdown on HCC cell proliferation was assessed by EdU assay. (G, H) The expression of PCNA in GCDH-overexpressing and knockdown tumor tissues by IHC assay. (I,J and K) The effect of GCDH overexpression and knockdown on HCC cell migration and invasion was assessed by transwell assay.


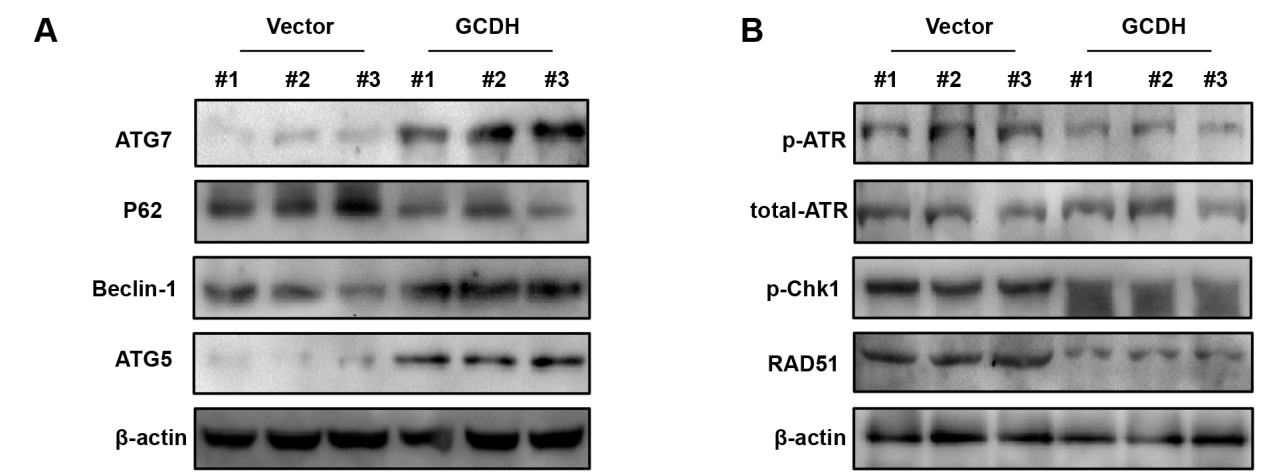


**Fig. S4.** (A) The expression of autophagy-related markers was analyzed by Western Blotting in vivo mouse xenograft model. (B) The expression of DNA damage repair markers was analyzed by Western Blotting in vivo mouse xenograft model.


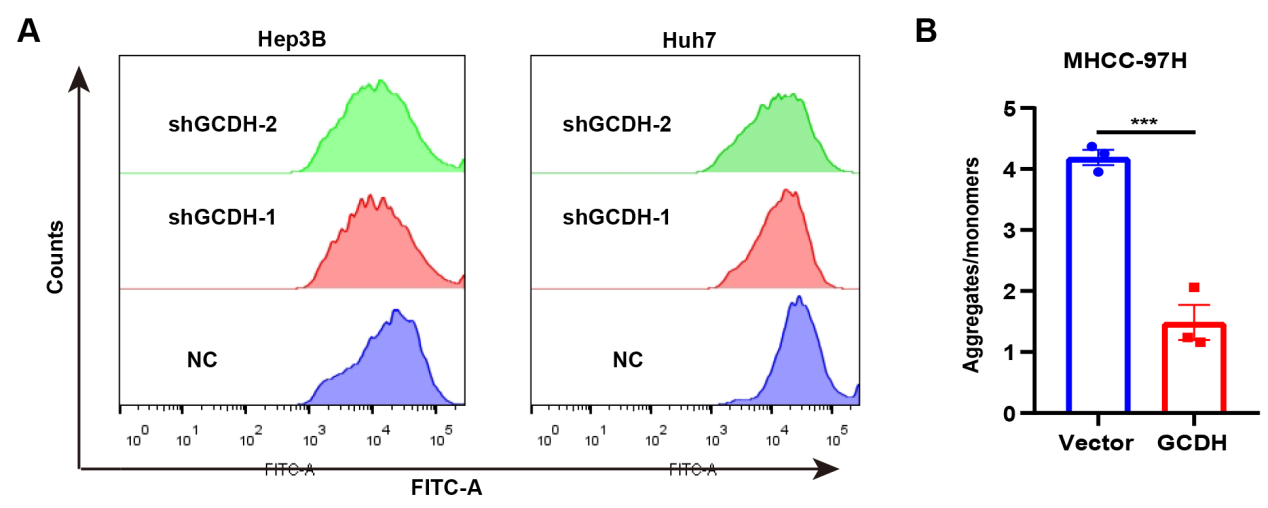


**Figure S5.** (A) The level of ROS was assessed by Flow cytometry in GCDH kn.ockdown HCC cells. (B) Mitochondrial membrane potential was determined by JC-1 staining. The ratio of aggregates/monomers was compared in the right bar graph.


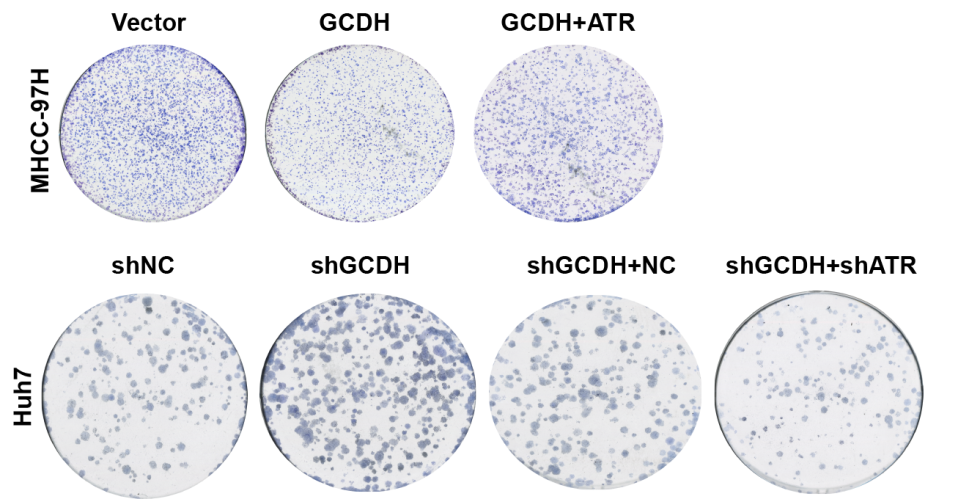


**Fig.S6.** The effect of ATR overexpression on GCDH overexpression HCC cell proliferation was assessed by colony formation assays.The effect of ATR knockdown on GCDH knockdown HCC cell proliferation was assessed by colony formation assays.


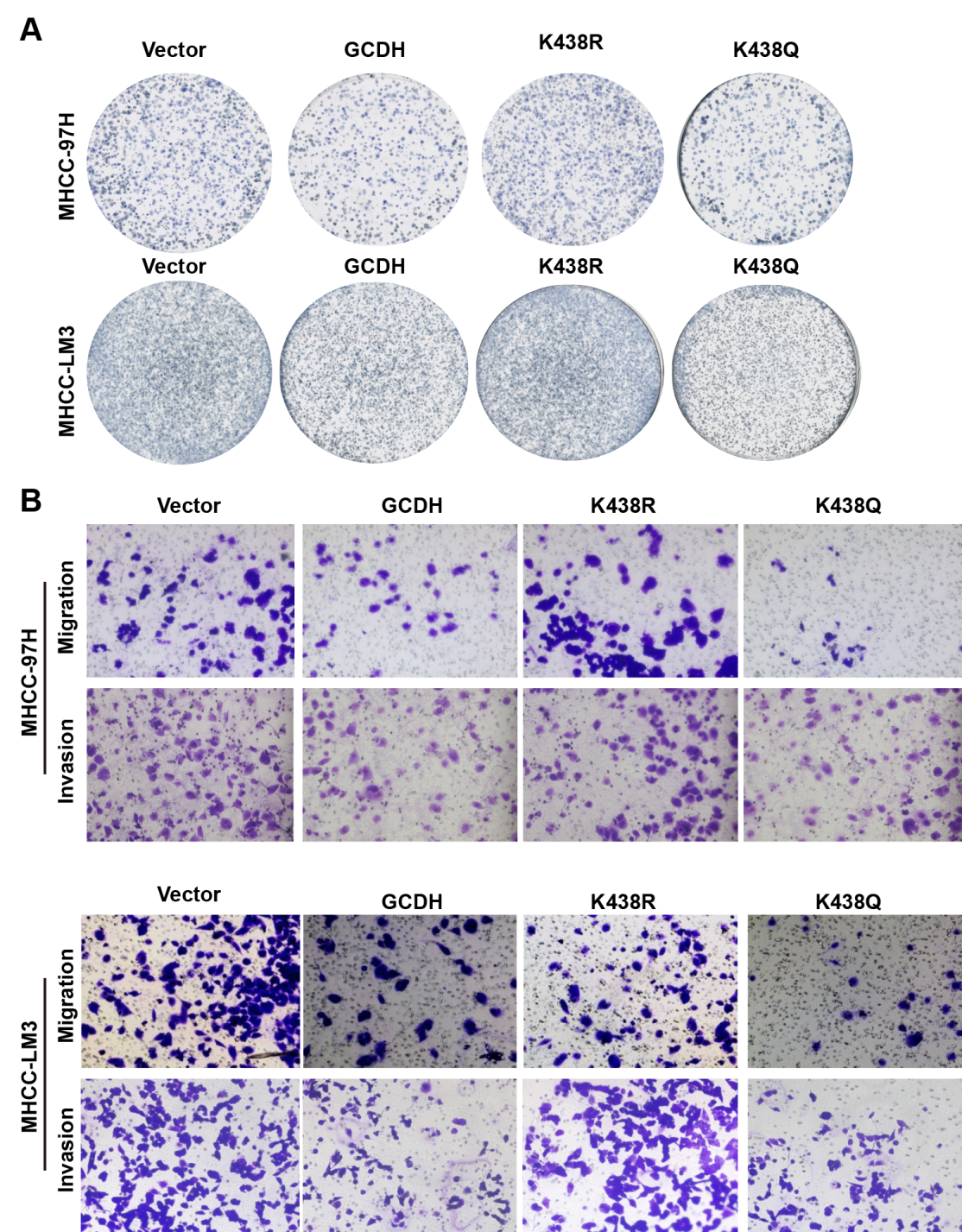


**Fig. S7.** (A) The effect of GCDH K438 acetylation on HCC cell proliferation was determined by colony formation assay. (B) The effect of GCDH K438 acetylation on HCC cell migration and invasion was determined by transwell assay.


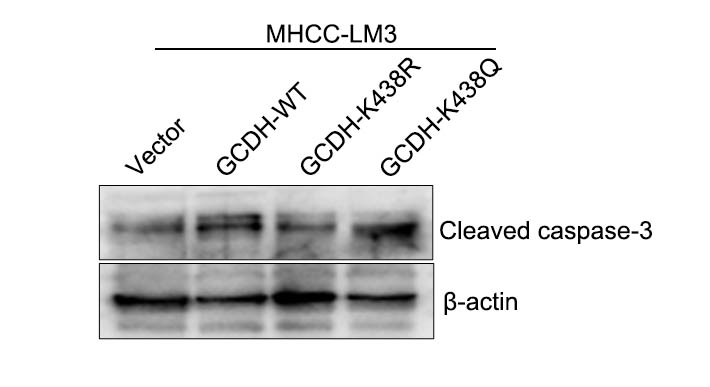


**Fig. S8.** The expression of cleaved caspase-3 was detected by WB in GCDH wild-type and acetylation-mimetic mutant-expressing MHCC-LM3 cells


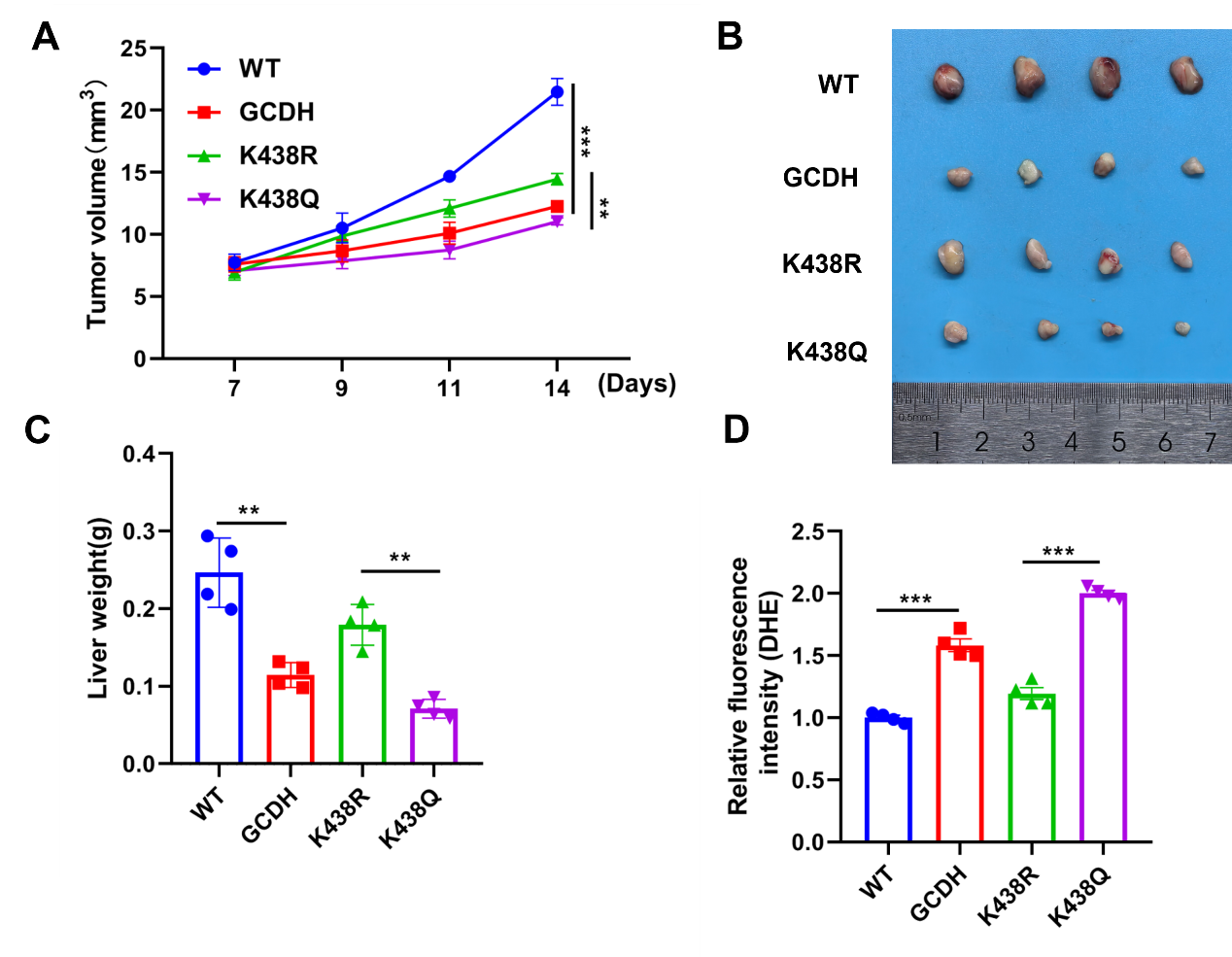


**Fig. S9.** MHCC-LM3 cells stably expressing both wild-type and acetylation-mimetic mutant forms of GCDH were injected subcutaneously into the flanks of nude mice. (A) Tumor growth was measured every 2 days. (B) Representative images of excised tumors are shown. (C) The dot plots show the results of the quantitative analysis of tumor weight. (D)The ROS level was examined in xenograft tissues.


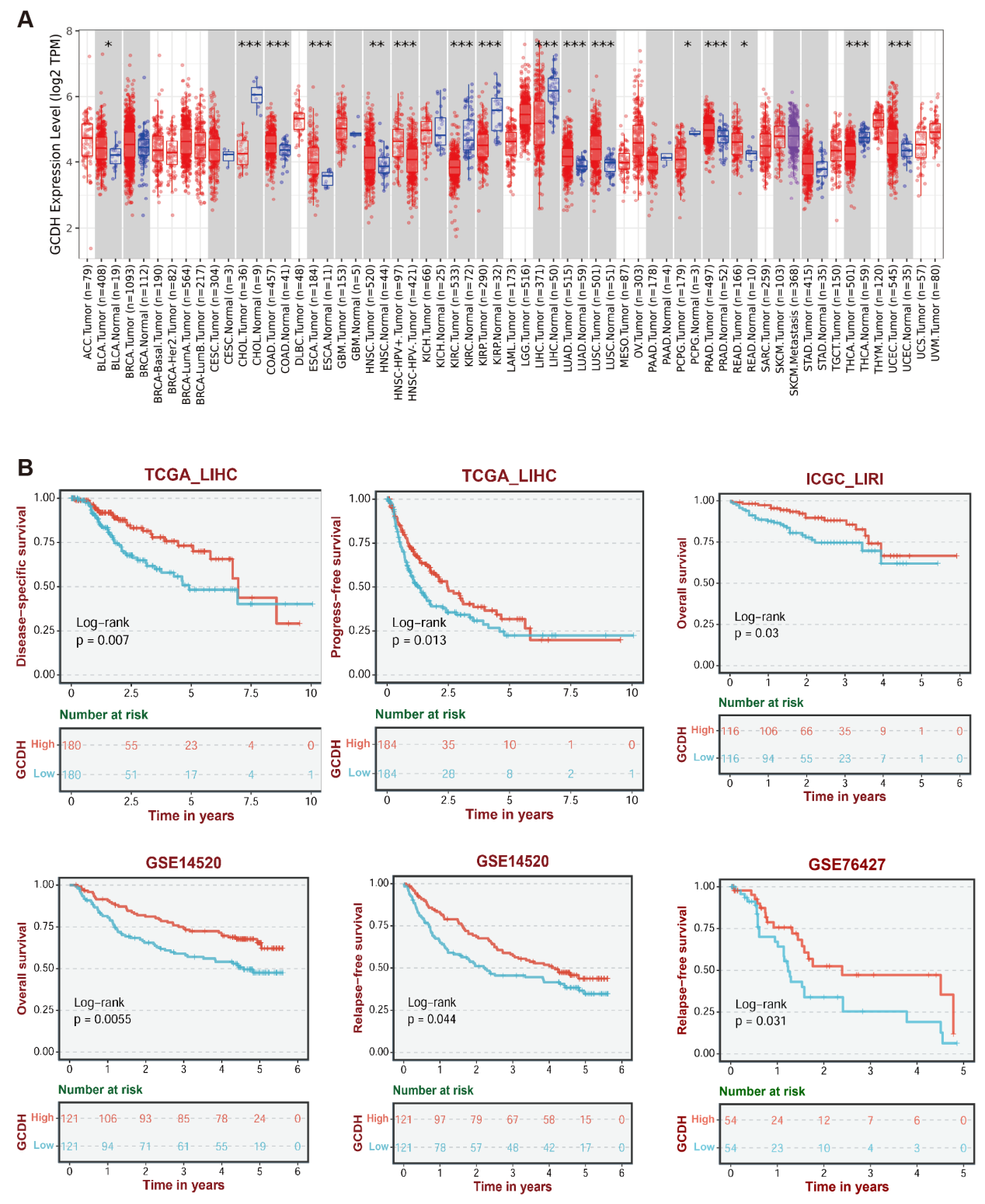


**Fig. S10.** (A) The expression of GCDH tumor tissues compared with paired corresponding noncancerous tissues was analyzed using data sets from TCGA. (B) Patients with low expression levels of GCDH shorter overall survival than patients with low expression levels, as determined using data sets from TCGA，GEO and ICGC.


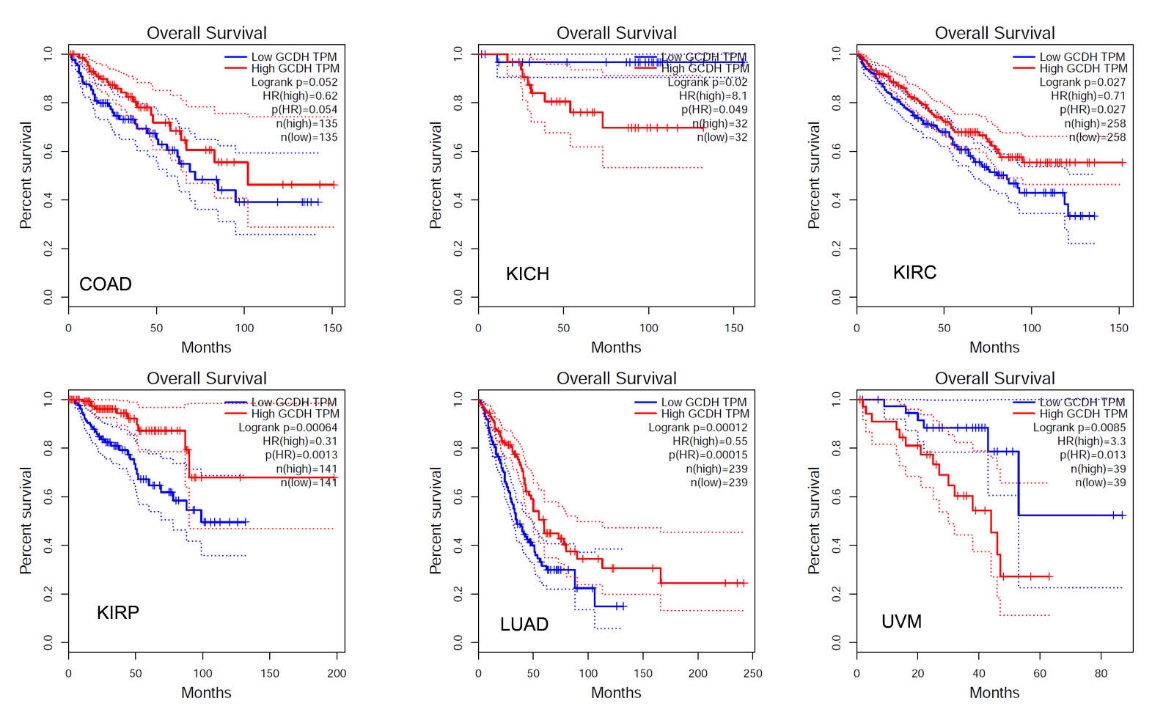


**Fig.S11.** Overall survival analysis of various tumors stratified by the GCDH expression using TCGA data.

**
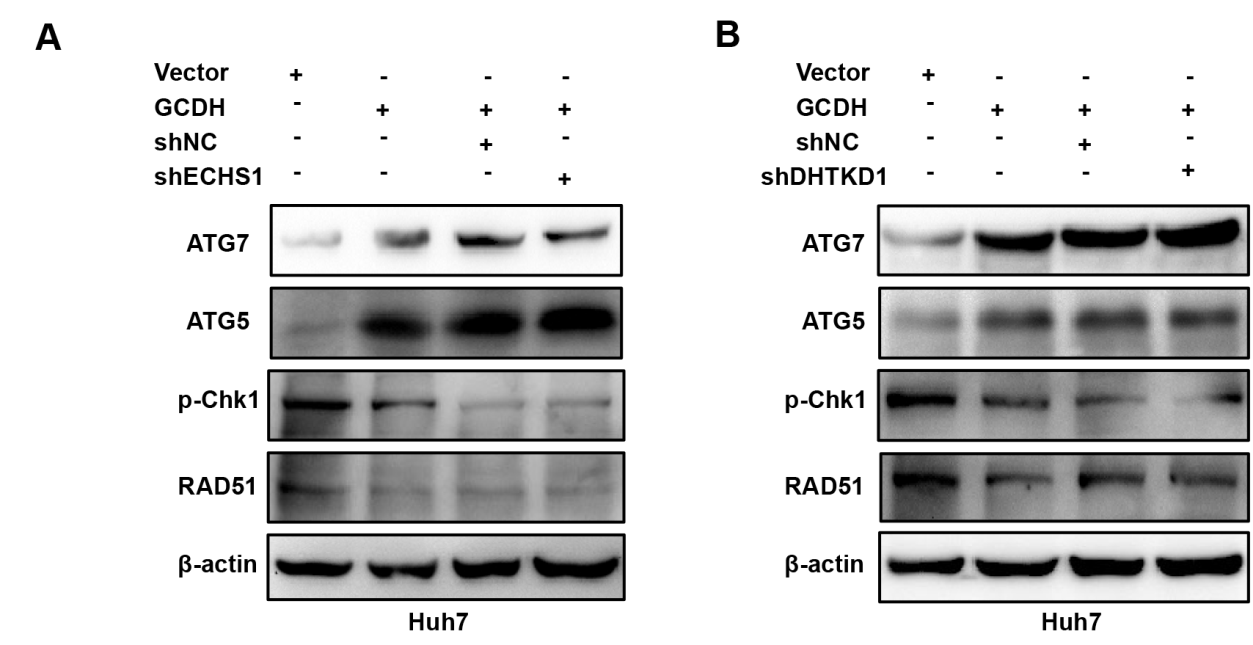
**

**Fig. S12.** The effect of other key enzymes in lysine metabolism on autophagy and DNA damage was assessed by Western Blotting.

**Supplementary Tables**

**Supplementary Table S1. The sequences of shRNA target**

| Identifier | Forward (5’-3’) |
| --- | --- |
| GCDH-1 | CGTTCAGATGTGTTCCTTAAA |
| GCDH-2 | CCACTACAACTCATCCAACAA |

**Supplementary Table S2. The sequences of gene-specific primers used for qRT-PCR.**

| Gene name | Forward (5’-3’) | | Reverse (5’-3’) |
| --- | --- | --- | --- |
| **Primers for qRT-PCR** | | |  |
| GCDH | | CCCTCGTCATGCACCCTATC | GGTCTTGGTCCCATTGAGGG |
| GAPDH | | AGAAGGCTGGGGCTCATTTG | AGGGGCCATCCACAGTCTT C |

**Supplementary Table S3. Antibodies used in this study**

| Antibody | Catalog | | Dilution | | | Company |
| --- | --- | --- | --- | --- | --- | --- |
| **For Western blotting** | | | | | | |
| GCDH | AV43559 | 1:1000 | | Sigma | | |
| Acetylated-Lysine | CST-9441 | 1:1000 | | CST | | |
| V5-Tag | ABT2170 | 1:1000 | | Abbkine | | |
| DYKDDDDK Tag | CST-14793 | 1:500 | | CST | | |
| P300 | sc-48343 | 1:200 | | Santa Cruz | | |
| HDAC1  p-ATR ser428  ATR | sc-81598  CST-2853  CST-2790 | 1:200  1:1000  1:1000 | | Santa Cruz  CST  CST | | |
| p-Chk1 ser345 | CST-2348 | 1:1000 | | CST | | |
| RAD51 | CST-8875 | 1:1000 | | CST | | |
| p-Histone H2AX | CST-9718 | 1:1000 | | CST | | |
| ATG9A | T55704 | 1:500 | | Abmart | | |
| ATG7 | T57051 | 1:500 | | Abmart | | |
| ATG5 | T55766 | 1:500 | | Abmart | | |
| LC3A/B | CST-12741 | 1:1000 | | CST | | |
| P62 | sc-48402 | 1:500 | | Santa Cruz | | |
| β-actin | A3854 | 1:10000 | | Sigma | | |
| Secondary antibody | HRP conjugated goat anti-rabbit IgG | 1:4000 | | Sigma | | |
| Secondary antibody | HRP conjugated goat anti-mouse IgG | 1:4000 | | Sigma | | |
| **For Immunohistochemistry** | | | | | | |
| GCDH | AV43559 | 1:200 | | | Sigma | |
| PCNA | Ab29 | 1:1000 | | | Abcam | |
| Secondary antibody | Envision kit (HRP, rabbit/mouse, DAB+) | Ready-to-use | | | DAKO | |
| **For Immunofluorescence staining** | | | | | | |
| GCDH | AV43559 | 1:500 | | | Sigma | |
| p-Histone H2AX | CST-9718 | 1:500 | | | CST | |
| P62 | sc-48402 | 1:50 | | | Santa Cruz | |
| p-ATR ser428 | CST-2853 | 1:200 | | | CST | |
| Secondary antibody | Alexa Fluor 594/488 anti-rabbit IgG | 1:50 | | | Invitrogen | |

**Supplementary Table S4.** Clinicopathological features of HCC patients (142 cases)

| Clinicopathological features | Number |
| --- | --- |
| **Age** |  |
| ＜55 | 54 |
| ≥55 | 88 |
| **Gender** |  |
| Male | 114 |
| Female | 28 |
| **Tumor size** |  |
| ≤ 5 cm | 53 |
| >5 cm | 89 |
| **AJCC grade** |  |
| I- II | 55 |
| III- IV | 87 |
| **Cirrhosis** |  |
| Negative | 48 |
| Positive | 94 |
| **AFP (ng/mL)** |  |
| ≤20 | 64 |
| >20  Missing | 75  3 |
| **HBV** |  |
| Negative | 31 |
| Positive | 111 |
| **MVI**  M0  M1  M2  Missing  **Capsule**  Negative  Positive  **Distant metastasis**  Negative  Positive | 50  50  41  1  71  71  139  3 |

**Supplementary Table S5.** Correlation between GCDH levels in HCC patients and their clinicopathological characteristics

| Clinicopathological features | Number | Low expression  N (%) | High expression  N (%) | *p* value |
| --- | --- | --- | --- | --- |
| **Age** |  |  |  |  |
| ＜55 | 54 | 28(51.9) | 26(48.1) | 0.203 |
| ≥55 | 88 | 36(40.9) | 52(59.1) |  |
| **Gender** |  |  |  |  |
| Male | 114 | 52(45.6) | 62 (54.4) | 0.793 |
| Female | 18 | 12(42.9) | 16(57.1) |  |
| **Tumor size** |  |  |  |  |
| ≤ 5 cm | 53 | 25(47.2) | 28(52.8) | 0.698 |
| >5 cm | 89 | 39(43.8) | 50(56.2) |  |
| **AJCC grade** |  |  |  |  |
| I- II | 55 | 27(49.1) | 28(50.9) | 0.444 |
| III- IV | 87 | 37(42.5) | 50(57.5) |  |
| **Cirrhosis** |  |  |  |  |
| Negative | 48 | 21(43.8) | 27(56.2) | 0.821 |
| Positive | 94 | 43(45.7) | 51(54.3) |  |
| **AFP (ng/mL)** |  |  |  |  |
| ≤20 | 64 | 23(35.9) | 41(64.1) | 0.040* |
| >20 | 75 | 40(53.3) | 35(46.7) |  |
| **HBV** |  |  |  |  |
| Negative | 31 | 14(45.2) | 17(54.8) | 0.991 |
| Positive | 111 | 50(45) | 61(55) |  |
| **Microvascular Invasion (MVI)** | | |  |  |
| M0 | 81 | 19(38) | 31(62) | 0.268 |
| M1  M2 | 9  41 | 27(54)  18(43.9) | 23(46)  23(56.1) |  |
| **Distant metastasis** | | |  |  |
| Negative | 139 | 62(44.6) | 77(55.4) | 0.425 |
| Positive | 3 | 2(66.7) | 1(33.3) |  |
| **Capsule** |  |  |  |  |
| Negative | 71 | 40(56.3) | 31(43.7) | 0.007** |
| Positive  **Tumor recurrence**  Negative  Positive | 71  49  41 | 24(33.8)  46(42.6)  18(52.9) | 47(66.2)  62(57.4)  16(47.1) | 0.290 |
